# Antioxidant Compounds in Microalgae and Cyanobacteria: A Sustainable Source of Bioactive Molecules

**DOI:** 10.3390/md24080288

**Published:** 2026-08-21

**Authors:** Patricia Gómez-Villegas, Encarnación Díaz-Santos, Rocío Rengel, Ana Molina-Márquez, José María Rodríguez-González, Javier Vigara, Rosa León, Antonio Leon-Vaz

**Affiliations:** 1 Departamento de Bioquímica Vegetal y Biología Molecular, Facultad de Química, Universidad de Sevilla, C. Prof. García González 1, 41012 Sevilla, Spain; 2 Laboratory of Biochemistry, Faculty of Experimental Sciences and Research Center in Natural Resources, Health and Environment (RENSMA), University of Huelva, 210071 Huelva, Spain; encarnacion.diaz@dqcm.uhu.es (E.D.-S.); rocio.rengel@dqcm.uhu.es (R.R.); ana.molina@dqcm.uhu.es (A.M.-M.); josemaria.rodriguez@dqcm.uhu.es (J.M.R.-G.); vigara@uhu.es (J.V.); rleon@uhu.es (R.L.); 3 Institute of Sustainable Processes, University of Valladolid, Dr. Mergelina, s/n, 47011 Valladolid, Spain; 4 Department of Chemical Engineering and Environmental Technology, University of Valladolid, Dr. Mergelina, s/n, 47011 Valladolid, Spain

**Keywords:** carotenoids, fatty acids, glutathione, phenolic compounds, ROS, terpenoids, vitamins

## Abstract

The search for antioxidant compounds from aquatic or marine environments to cope with the harmful effects of reactive oxygen species (ROS) is one of the major challenges in contemporary blue biotechnology. Among marine microorganisms, microalgae have emerged as promising candidates for the discovery and production of natural bioactive molecules with antioxidant properties. This review highlights the potential of microalgae and cyanobacteria as a sustainable source of antioxidant compounds and examines their growing relevance in biotechnology and pharmaceutical applications. A broad range of antioxidant metabolites produced by microalgae, including carotenoids, fatty acids, vitamins, polyphenols, and flavonoids, has also been discussed, with particular emphasis on their antioxidant mechanisms and bioactive properties. This review also integrates antioxidant mechanisms with the physiological and metabolic responses, underlying antioxidant production and sustainable strategies used to enhance their accumulation. Furthermore, microalgae offer the advantage of sustainable production systems, with the potential to enhance the biosynthesis and accumulation of valuable compounds through optimized cultivation strategies. Thus, different sustainable approaches aimed at increasing antioxidant compound production or reducing operational costs in microalgae cultivation are discussed to identify efficient and economically viable processes that maximize the biotechnological potential of these microorganisms for future industrial applications.

## 1. Introduction

Oxidative stress is defined as an imbalance between reactive oxygen species (ROS) production and the antioxidant defense system. This equilibrium is essential for cellular homeostasis, and its disruption can lead to serious cellular damage due to the accumulation of ROS, including hydrogen peroxide (H_2_O_2_), superoxide radicals (O_2_^•−^), or hydroxyl radicals (·OH), among others, ultimately resulting in oxidative stress [1,2]. Excessive and prolonged ROS production damages macromolecules, such as proteins, lipids, DNA; disrupts cellular structures; and impairs physiological functions. These effects in the human population contribute to the development and progression of numerous non-communicable diseases (NCDs) [3]. Cells have developed several antioxidant mechanisms to counteract ROS accumulation. Different enzymes, including superoxide dismutase (SOD), catalase (CAT), glutathione peroxidase (GPx), or ascorbate peroxidase (APX), have been reported to play a central role in ROS detoxification [4,5,6]. Furthermore, organisms can also produce non-enzymatic responses to ROS accumulation by synthesizing molecules with high antioxidant capacity, which also helps to maintain the redox balance [7]. The production of these molecules from natural sources has received increasing attention in recent years. Natural products obtained by microorganisms account for more than one-third of new FDA-approved molecular products. Many of these compounds exhibited high anti-inflammatory, antibiotic, antiviral, or cytotoxic activity, underscoring the potential of these molecules, which were obtained from natural sources [8]. Thus, the exploration of bioactive molecules produced by microorganisms has become a prominent focus in biotechnology research.

**Figure 1:**
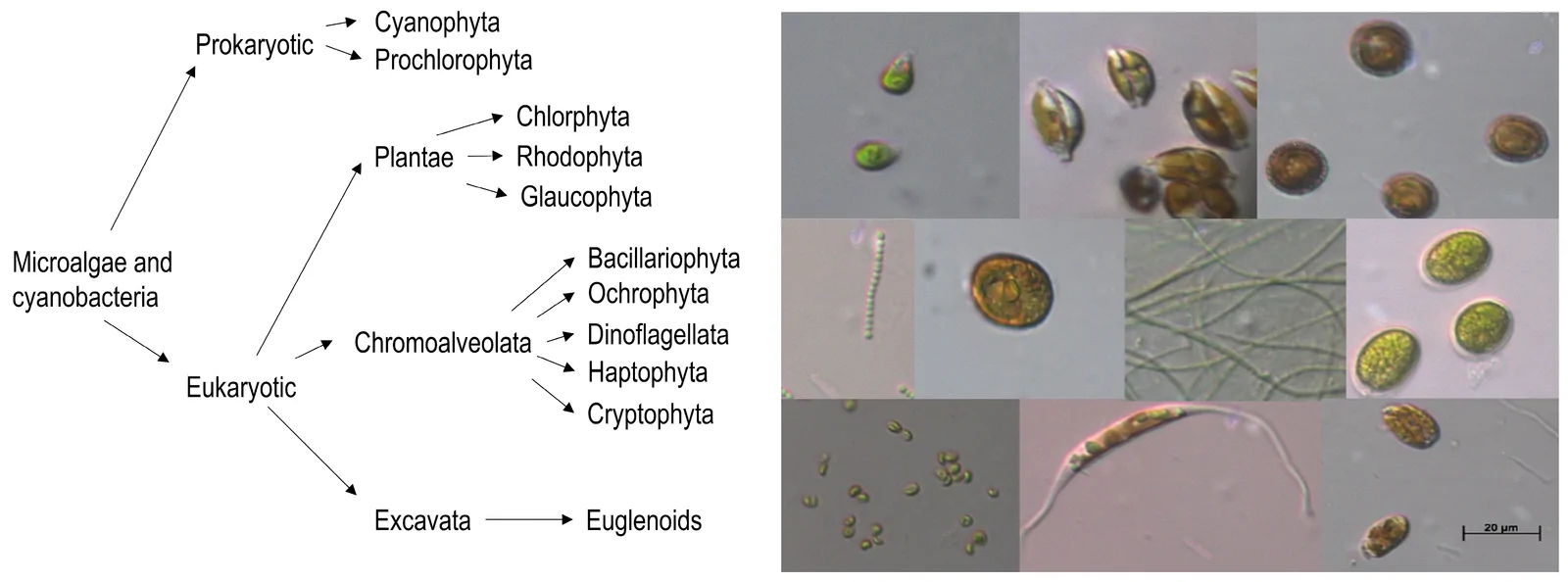
Taxonomical classification of microalgae and cyanobacteria and microscopical pictures of microalgae kindly provided by the Institute of Marine Sciences of Andalusia (Spain).

Microalgae and cyanobacteria are a diverse group of photosynthetic organisms with high biotechnological potential. This diverse group of microorganisms includes both prokaryotic and eukaryotic lineages with diverse cellular organization, secondary metabolites composition, and metabolic capabilities (Figure 1). Although cyanobacteria are taxonomically distinct from eukaryotic microalgae, they can be included within the broader microalgal context as the main prokaryotic lineage because of their photosynthetic metabolism, microscopic nature, and comparable biotechnological applications as sources of bioactive compounds in the context of this review. On the other hand, eukaryotic microalgae encompass diverse groups such as green microalgae, diatoms, dinoflagellates or other lineages (Figure 1). Moreover, these microorganisms have demonstrated a high adaptation capacity to different habitats, which include rivers, polar lakes, marine seas, hot springs, or even desert sand [9]. Their capacity to thrive in extreme ecosystems makes them one of the most versatile microorganisms, with unique survival mechanisms to withstand harsh conditions and to balance the increase in ROS [10]. One of the main advantages of microalgae is their high capacity to reduce CO_2_ levels through photosynthesis. These microorganisms capture and reduce CO_2_ 10–50 times more efficiently than terrestrial plants, being able to fix about 50 gigatons of CO_2_ per year worldwide [11,12]. Moreover, microalgal growth does not compete for land resources; they require minimal nutritional requirements, and the CO_2_ fixed can be metabolically transformed into energy and microalgal biomass [13,14]. All these advantages make microalgae and cyanobacteria cultivation a sustainable approach to address environmental problems, such as mitigating global emissions and achieving carbon neutrality.

These microorganisms not only fix high amounts of CO_2_ but also possess a broad range of biotechnological applications. Different studies have demonstrated that microalgae can simultaneously use organic and inorganic pollutants during wastewater reclamation processes with CO_2_ sequestration from flue gas, underscoring their environmental relevance [15,16,17]. These findings highlight the sustainability and multifunctionality of microalgal cultivation processes. In addition to CO_2_, total organic carbon, nitrogen, or phosphorus, microalgae can also remove other persistent pollutants from wastewater, including heavy metals, organic pollutants, and pesticides [18,19,20].

**Figure 2:**
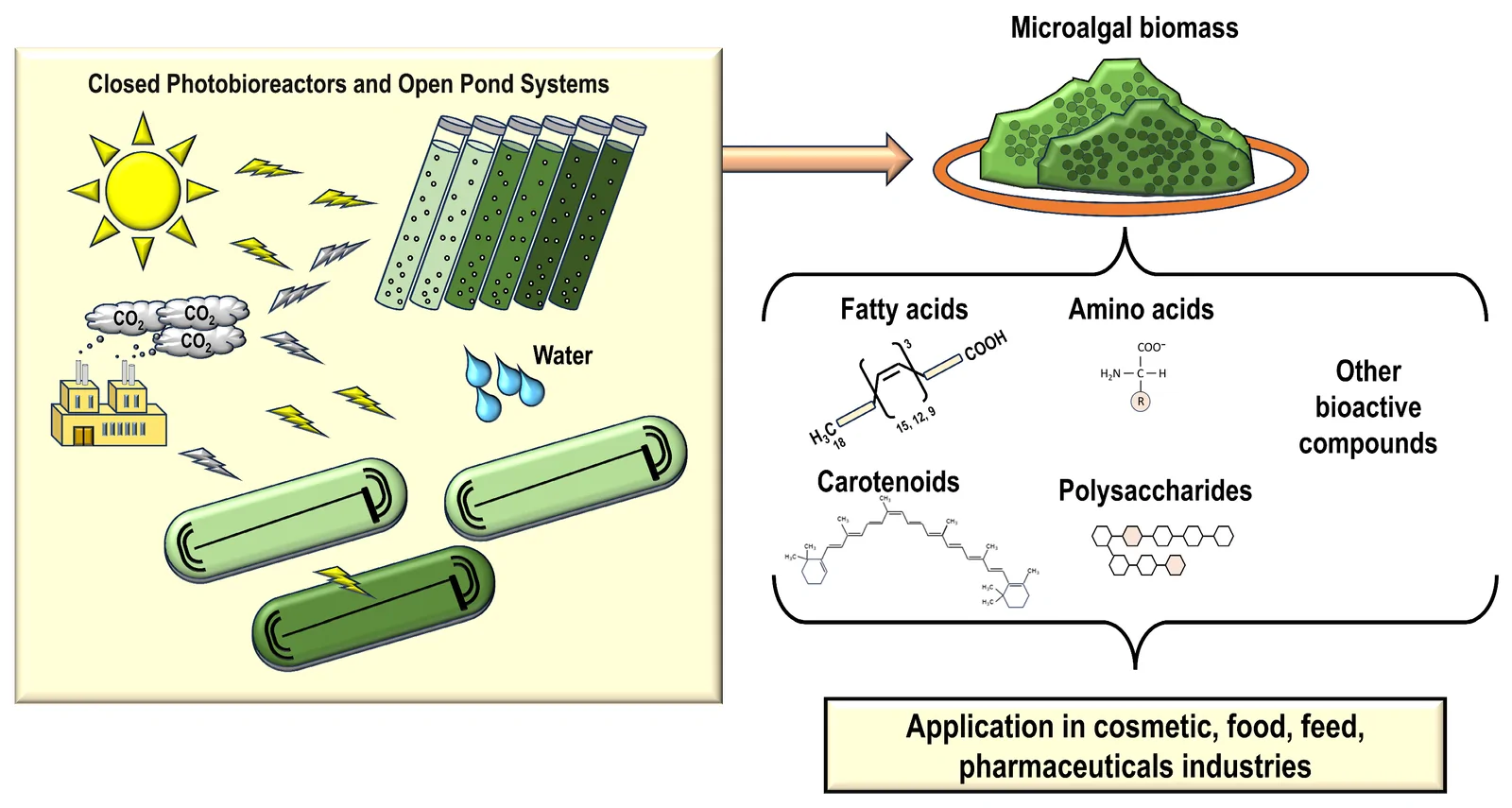
Production of bioactive compounds by microalgae.

Beyond their environmental applications, microalgae can also produce a variety of high-value compounds used in the cosmetic, food, feed, and pharmaceutical industries. The global market of microalgae products is valued at approximately US $4.96 billion and is expected to increase up to US $9.1 billion in 2032 [21]. In recent decades, numerous microalgal species have been investigated because of their ability to produce these bioactive compounds, which include fatty acids [22], carotenoids [23], amino acids [24], or polysaccharides [25], highlighting their wide range of valuable molecules (Figure 2). The production of these metabolites can be further enhanced by modifying culture conditions or by applying genetic engineering strategies, thereby improving the economic feasibility of their biotechnological exploitation [26,27,28].

Among the variety of compounds produced by microalgae, some of them have demonstrated a remarkable capacity to cope with ROS, showing their antioxidant and bioactive potential. Primary metabolites, such as fatty acids or amino acids, as well as secondary metabolites, including carotenoids, vitamins, phenolic compounds, or flavonoids, have all demonstrated significant antioxidant capacity [29,30]. This activity can be addressed through different analytical assays. Among the available antioxidant assays, the most widely used in the literature is the 1,1-diphenyl-2-picrylhydrazil (DPPH) radical scavenging assay, which measures the ability of a compound to reduce the DPPH radical in the presence of a radical scavenger [31]. Other commonly applied assays include the 2,2-azino-*bis*-3-ethylbenzothiazoline-6-sulphonic acid (ABTS) assay, which measures the capacity to scavenge synthetic organic radicals through electron donation; oxygen radical absorbance capacity (ORAC), which quantifies total antioxidant capacity by measuring how effectively a sample prevents the oxidative degradation of a fluorescent probe by peroxyl radicals, expressed in Trolox equivalents; thiobarbituric acid reactive substances (TBARSs), which quantify lipid peroxidation; total antioxidant capacity (TAC), which measures the combined antioxidant power of all compounds present in a sample [32,33,34,35]; and ferric reducing antioxidant power (FRAP), which measures the specific ability to reduce a ferric complex (Fe^3+^) to its ferrous form (Fe^2+^) by evaluating the electron-donating capability [36]. Consequently, results obtained from these diverse methods are not directly comparable because they rely on different reaction mechanisms and experimental conditions. Numerous studies have included these methods to evaluate the antioxidant capacity of microalgal extracts; however, their results vary considerably, primarily due to these distinct mechanisms, as well as the high variability in extraction methods, solvents, concentrations (microalgal extracts and standards), or reaction times between studies [37,38,39,40,41,42]. In this context, some of the studies have been able to establish a relationship between the content of specific bioactive compounds in microalgae and the antioxidant activity tested in the extracts [43,44,45]. However, other studies have reported no clear correlation between the abundance of these compounds and the outcomes of antioxidant assays [42,46,47]. This lack of agreement suggests that microalgal extracts contain numerous molecules with antioxidant properties and that a previous purification step is required to accurately determine such relationships.

Thus, this review assesses the potential of microalgae to produce molecules with antioxidant activity. Moreover, it discusses the main challenges that must be addressed to obtain these compounds in a sustainable manner, highlighting the potential of these microorganisms as an environmentally sustainable source of antioxidant molecules from the pharmaceutical, feed, and food industries. Recent reviews have discussed microalgae as sources of bioactive compounds, including antioxidants and those with antimicrobial properties, and have addressed their nutritional, health-promoting or food-related applications [48]. However, these studies consider antioxidant compounds as part of broader bioactive composition of microalgal biomass or focus on specific application areas. In contrast, the present review provides an integrating analysis of microalgal antioxidants by connecting the main classes with their antioxidant mechanisms, the physiological and metabolic responses, and the cultivation strategies used to enhance their production. Finally, this review discusses the downstream processing and sustainable production of these compounds, thereby linking antioxidant functionality with the technological challenges that limit their industrial exploitation. For the development of this review, a literature search was conducted to identify relevant publications addressing antioxidant compounds produced by microalgae and cyanobacteria. The databases used were Web of Science, Scopus and PubMed, covering publications between 2019 and 2026 using combination of keyworks including “microalgae”, “cyanobacteria”, “antioxidant compounds”, “carotenoids”, “fatty acids”, “vitamins”, “polyphenols”, “sustainability”, and “biotechnology”. Relevant earlier studies were also included when considered for a relevant discussion. Publications were selected based on their relevance to the production strategies, mechanisms, or biotechnological applications of antioxidant compounds. Publications directly related to these topics were included, whereas those out to the scope of this review or lacking sufficient relevant information were excluded.

## 2. Antioxidant System in Microalgae

**Figure 3:**
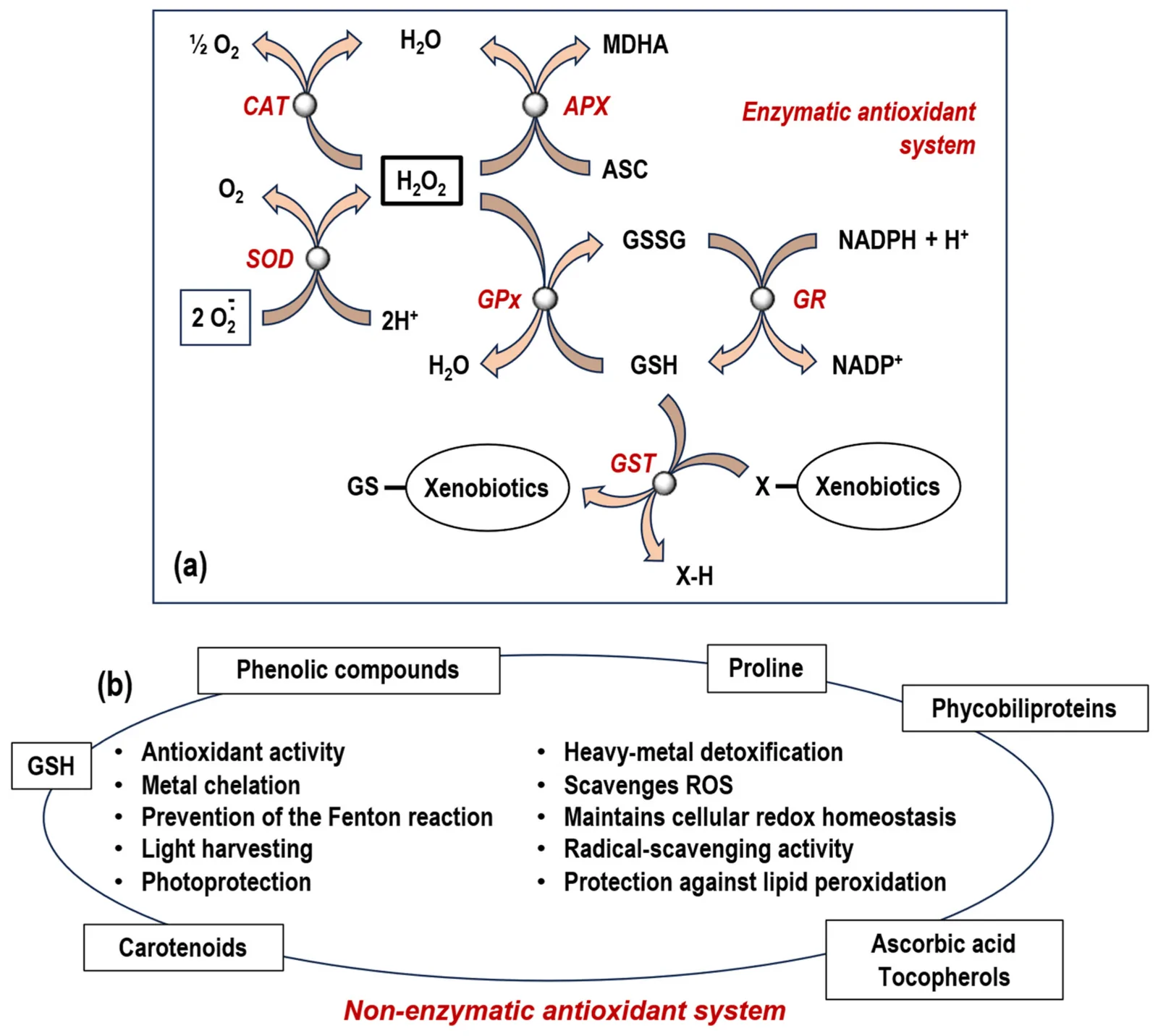
Main components of the antioxidant systems in microalgae: (**a**) enzymatic antioxidant system; (**b**) non-enzymatic antioxidant system.

Microalgae have evolved a sophisticated and multifaceted antioxidant defense system to counteract the damaging effects of oxidative stress. These photosynthetic microorganisms inhabit diverse and often extreme ecological niches and are frequently exposed to fluctuating environmental stressors, such as high solar irradiance, extreme temperatures, and nutrient fluctuations, which promote the intracellular accumulation of ROS, in addition to those already generated during photosynthesis as normal metabolic byproducts. To maintain cellular redox homeostasis and protect vital macromolecules, such as DNA, proteins, and membrane lipids, from oxidative damage, microalgae employ a synergistic network of antioxidant enzymes and non-enzymatic metabolites (Figure 3) [49]. This complex defensive machinery not only ensures survival under adverse conditions but also determines the physiological fitness and biotechnological potential of microalgae, with different species producing unique combinations under various stresses (light, metals, and nutrients).

### 2.1. Enzymatic Antioxidant System

The primary line of defense against ROS-induced damage is the enzymatic antioxidant system, which operates by directly neutralizing and detoxifying superoxide radicals and hydrogen peroxide into stable molecules. Unlike non-enzymatic metabolites, which are often consumed during the scavenging process, these enzymes act catalytically, allowing the cell to respond rapidly to acute oxidative bursts. The efficiency of this system relies on a coordinated cascade of enzymes primarily located in the chloroplasts and mitochondria. The microalgal enzymatic detoxification system comprises key antioxidant enzymes, including superoxide dismutase (SOD), catalase (CAT), ascorbate peroxidase (APX), glutathione peroxidase (GPx), glutathione reductase (GR), and glutathione-S transferase (GST) [50].

The metalloenzyme superoxide dismutase constitutes the first line of defense, converting the highly reactive superoxide radical (O_2_^•−^) and hydrogen peroxide (H_2_O_2_) into oxygen (O_2_). Three major isozymes differing in their protein folding and metal cofactors are reported in microalgae: Mn-SOD, typically localized in the mitochondria, Fe-SOD, primarily found in the chloroplast stroma, and Cu/Zn-SOD, found in the cytosol and thylakoid membranes of certain species of green algae. Microalgae naturally produce SOD, with certain species like *Tetraselmis chuii* being rich sources, leading to their use in supplements like TetraSOD^®^ to enhance antioxidant defense, reduce inflammation, and improve cellular health in humans [51]. SOD activity increases under various stress conditions. While heavy metals like copper or cadmium induce SOD production in *Chlorococcum* sp. [52], iron and salinity stresses have been shown to significantly enhance SOD activity in *Pseudochlorella pringsheimii* [53].

Catalase is a heme-containing tetrameric protein crucial for the rapid removal of H_2_O_2_, converting it into water and molecular oxygen without requiring a reducing equivalent. Catalase is particularly active in peroxisomes during photorespiration and is also present, to a lesser extent, in the chloroplast, cytosol, and mitochondria. Recent research focused on CAT as a primary biomarker for heavy metal toxicity. Studies on the acidophilic microalga *Coccomyxa onubensis* have demonstrated that CAT activity is essential for its high tolerance to arsenic [54].

Two types of peroxidase enzymes are present in microalgae, namely ascorbate peroxidase and glutathione peroxidase, which reduce H_2_O_2_ using ascorbate and glutathione, respectively, as electron donors. Moreover, peroxiredoxins (Prxs) further assist in reducing hydroperoxides, protecting the integrity of photosynthetic membranes [55]. In summary, microalgal antioxidant enzymes eliminate ROS by converting them into H_2_O_2_, which is then converted into H_2_O, helping preserve cellular structure and function.

The ascorbate–glutathione (Asada–Halliwell) cycle is the most vital pathway for H_2_O_2_ scavenging in the chloroplasts of microalgae. It involves a series of redox reactions where ascorbate peroxidase reduces H_2_O_2_ using ascorbate as an electron donor, while glutathione reductase recycles oxidized glutathione to maintain the cellular redox state [56]. In industrial microalgae production (for biofuels or supplements), this cycle is a “bottleneck”. Therefore, some studies are currently focusing on overexpressing genes like *APX* or *GR* in genera like *Chlorella* and *Nannochloropsis* to create strains that can grow under higher light and lower temperatures, resisting oxidative stress. In addition, this cycle is a critical metabolic switch that dictates whether a cell stays in its green vegetative state or begins producing high-value secondary pigments like astaxanthin and beta-carotene. This relationship is particularly vital for industrial species like *Haematococcus lacustris* and *Dunaliella salina*. Some studies center on the addition of small doses of hydrogen peroxide or the slight inhibition of GR to reach the overproduction of both the enzymes of the Asada–Halliwell cycle and high-value pigments simultaneously [57].

Glutathione reductase is a critical enzyme in the cellular antioxidant defense system. This flavoenzyme functions as a homodimer, maintaining the pool of reduced glutathione (GSH), which protects cells from oxidative damage. This enzyme catalyzes the reduction of oxidized glutathione (GSSG) back to its active reduced form (GSH), with NADPH as a reducing cofactor to provide the necessary electrons. Maintaining a high GSH/GSSG ratio ensures that the cell remains in a reducing environment, which is essential for neutralizing free radicals and ROS [58]. When microalgae are exposed to metals, GR activity typically spikes to ensure a continuous supply of GSH for both metal binding and the repair of metal-induced lipid peroxidation [59]. In species such as *Dunaliella salina*, GR activity increases to maintain redox homeostasis when the cell is dehydrated or exposed to high salt concentrations [60]. In addition, GR helps manage nutrient limitation when nitrogen or phosphorus is scarce, preventing the resulting oxidative burst from killing the cell. The GSH/GSSG ratio maintained by GR acts as a redox signaling for the microalga, regulating the activation of stress-response genes or slowing down the cell cycle [61].

Glutathione S-transferase (GST) is part of a diverse family of metabolic enzymes that play a crucial role in the detoxification process. Unlike GR, which recycles glutathione, GST uses reduced glutathione (GSH) to neutralize toxic molecules. Its core functions in microalgae include the conjugation of xenobiotics, making the toxins more water-soluble and allowing the cell to either export them or sequester them safely in the vacuole, and the processing of organic hydroperoxides and byproducts of lipid peroxidation caused by exposure to metals [62]. GST expression is heavily upregulated in response to high UV radiation, extreme temperatures, and salinity shifts, reducing the resulting cellular damage. Studies in *Chlamydomonas reinhardtii* have identified specific GST classes (such as Sigma and Phi) that are uniquely adapted to handle the oxidative stress generated during photosynthesis [63]. In addition, GST activity is widely used as a biomarker in aquatic toxicology to indicate the presence of organic pollutants or heavy metals in the water [64]. Finally, some studies are focusing on engineering microalgae with overexpressed GST genes to create biological filters capable of cleaning contaminated industrial wastewater [53].

It is important to highlight that the microalgal enzymatic antioxidant system operates as an interconnected network rather than a series of isolated pathways. SOD acts as the first line of defense, converting superoxide radicals (O_2_^•−^) into hydrogen peroxide (H_2_O_2_). This intermediate is subsequently cleared by complementary downstream mechanisms: CAT manages bulk H_2_O_2_ detoxification, while GPx and APX target trace concentrations, particularly within chloroplasts and mitochondria. Crucially, APX efficiency relies on GR to regenerate the GSH pool via the ascorbate–glutathione cycle. This same GSH pool is simultaneously utilized by GST to detoxify lipid peroxides. Ultimately, microalgal survival depends on the kinetic synchronization of this entire cascade, where upstream ROS production is strictly balanced by downstream clearance.

### 2.2. Non-Enzymatic Antioxidant System

In addition to the enzymatic machinery, microalgae synthesize a diverse array of non-enzymatic low-molecular-weight compounds. These molecules act as direct scavengers of ROS, quenchers of singlet oxygen, and chain breakers that prevent the propagation of lipid peroxidation. The accumulation of these metabolites is often a secondary response to environmental stress, providing an additional layer of protection for the cell. These compounds include carotenoids, phycobiliproteins, phenolic compounds, vitamins, reduced glutathione (GSH), proline, hydroquinones, and polyamines.

Carotenoids are the most prominent non-enzymatic antioxidant metabolites in microalgae. They are integrated into thylakoid membranes and serve two main functions: light harvesting and photoprotection. Primary carotenoids (e.g., lutein and β-carotene) are essential for the structural integrity of the photosynthetic apparatus, while secondary carotenoids (e.g., astaxanthin and canthaxanthin) are produced in massive amounts under stress such as nutrient deprivation and salinity stress [65]. Pigments like astaxanthin (from *Haematococcus lacustris*), β-carotene (from *Dunaliella salina*), and lutein are highly effective at quenching singlet oxygen and scavenging free radicals [66]. They assist in photoprotection, membrane regulation, and the stabilization of light-harvesting complexes, contributing ~20–30% of the absorbed light energy and broadening the photosystem’s absorption spectrum [67]. Moreover, the stored carotenoids can be utilized by microalgal cells as energy and carbon sources to reactivate their metabolism under less stressful conditions [68]. In this sense, the xanthophyll cycle is a rapid response mechanism involving the interconversion of xanthophyll pigments to dissipate excess light energy as heat. In green algae and higher plants, this process involves the reversible conversion of violaxanthin to antheraxanthin and zeaxanthin, whereas in diatoms and other chromophyte algae, it is mediated by the reversible conversion of diadinoxanthin to diatoxanthin [69,70].

Phycobiliproteins are unique to cyanobacteria, rhodophyta (red algae), and cryptomonads. These water-soluble pigments are divided into three main types, namely allophycocyanin, phycocyanin, and phycoerythrin, according to their spectral properties; exhibit strong radical-scavenging activity; and have gained significant attention for their therapeutic and anti-inflammatory properties [71].

Phenolic compounds, including flavonoids and tannins, are potent antioxidants due to their ability to donate electrons or hydrogen atoms to free radicals. They also act as metal chelators, preventing the Fenton reaction, which generates the highly toxic hydroxyl radical (OH^•^) [72]. Several microalgal species accumulate phenolic compounds under harsh environmental conditions, enhancing their antioxidant capacity [73].

Water-soluble ascorbic acid (vitamin C) and lipid-soluble tocopherols (vitamin E) protect cellular membranes from lipid peroxidation by donating electrons to stabilize radicals. Among the tocopherols, which are mainly located in the thylakoid membranes, α-tocopherol is the most common form in microalgae. It is a major lipid-soluble antioxidant that protects polyunsaturated fatty acids (PUFAs) from lipid peroxidation by neutralizing lipid peroxyl radicals [74].

Glutathione (GSH) is a critical thiol tripeptide that serves as an electron donor and a cofactor for various detoxification enzymes. It is present in nearly all cellular compartments and plays a crucial role in various cellular processes, including stress-responsive gene expression. Its high reductive potential, driven by a nucleophilic cysteine residue, enables it to scavenge ROS such as H_2_O_2_ and singlet oxygen, protecting biomolecules through glutathiolation or reduction [75]. GSH also contributes to heavy metal detoxification, maintaining cellular redox homeostasis [76,77].

Certain amino acids, such as proline, also possess antioxidant properties. The proposed antioxidant protective mechanisms include the stabilization of proteins and antioxidant enzymes, direct ROS scavenging, and the regulation of intracellular redox homeostasis by inducing GSH accumulation and modulating cellular signaling [78].

Lipophilic hydroquinones, such as ubiquinol and plastoquinol, are structurally integrated into microalgal cellular and thylakoid membranes, where they act as critical electron donors. These molecules operate in continuous redox cycles, directly neutralizing lipid peroxyl radicals to suppress oxidative chain reactions and preserve the integrity of the photosynthetic machinery [79]. Concurrently, some microalgae accumulate aliphatic polyamines—predominantly putrescine, spermidine, and spermine—which function as positively charged molecular shields [80]. At physiological pH, these polycations bind electrostatically to the negatively charged phosphate heads of membrane phospholipids and nucleic acids, physically stabilizing the lipid bilayer against lipid peroxidation under severe abiotic stress, such as heavy metal contamination or high salinity. Furthermore, polyamines act as signaling modulators within the microalgal cytosol, triggering the upregulation of key antioxidant enzymes like SOD and CAT, while inducing the synthesis of GSH to restore global cellular redox homeostasis [81].

Overall, the antioxidant system of microalgae is a highly coordinated network that integrates enzymatic and non-enzymatic components to ensure physiological resilience, as summarized in Figure 3. Understanding the molecular triggers of these defenses not only provides insights into algal ecology but also offers a developmental pathway for optimizing the commercial production of natural antioxidants. Microalgae are rich and sustainable sources of these potent natural antioxidants, making them valuable for food, cosmetics, and medicine as alternatives to synthetic compounds. Some species (like *Chlorella*, *Dunaliella*, and *Arthrospira*) produce different antioxidants in response to environmental stressors like UV light or nutrient deprivation.

## 3. Antioxidant Compounds Produced by Microalgae

### 3.1. Carotenoids

Carotenoids represent a class of significant antioxidant isoprenoid compounds produced by microalgae, playing a direct role in the processes of photosynthesis, protection against photooxidative stress, and the generation of high-value biomass. These pigments comprise hydrocarbon carotenoids, such as β-carotene and oxygenated derivatives, or xanthophylls, including lutein, zeaxanthin, violaxanthin, astaxanthin, and fucoxanthin, among many others [82]. The carotenoid profile is influenced by light and other environmental conditions and varies between microalgal groups, making it an important taxonomic marker. Green microalgae are important sources of β-carotene, lutein and astaxanthin, while fucoxanthin is particularly associated with diatoms and other ochrophyte microalgae [82,83,84].

In several species of microalgae, the accumulation of carotenoids can be enhanced by manipulating various operational factors, including light intensity, nutrient availability, salinity, and cultivation techniques [83]. The observed physiological plasticity has facilitated the development of production systems for natural β-carotene and astaxanthin. This strategy is currently being investigated to produce lutein and fucoxanthin with the utilization of cultivation optimization and strain engineering techniques [83,85,86].

#### 3.1.1. Metabolic Importance of Carotenoids

Carotenoids are essential components of the photosynthetic apparatus of microalgae. The biosynthesis of carotenoids in these organisms begins with the synthesis of phytoene from geranylgeranyl diphosphate, followed by a series of desaturation, isomerization, cyclization, and oxygenation reactions. These reactions generate different carotene and xanthophyll branches [82]. In the context of photosynthetically active cells, carotenoids have been observed to be associated with thylakoid membranes and pigment–protein complexes, contributing to light harvesting, energy transfer, and the structural stability of the antenna and reaction centers [82,87].

In environments where light absorption exceeds the capacity of photosynthetic electron transport and carbon fixation, the photoprotective function of carotenoids becomes particularly significant. In such conditions, the excitation energy has been shown to promote the formation of chlorophyll triplet states and singlet oxygen [65,82]. This, in turn, has been demonstrated to lead to the oxidation of membrane lipids, proteins, and pigments. Carotenoids have been shown to reduce this damage through several mechanisms, including physical quenching of singlet oxygen, deactivation of excited chlorophyll states, and participation in mechanisms of non-photochemical quenching [88].

The contribution of carotenoids to photosynthetic protection varies among microalgal lineages. The presence of distinct xanthophyll-cycle pigments, as well as the differential accumulation of constitutive or stress-induced carotenoids, has been demonstrated to reflect this diversity. It is this differential accumulation that determines both the physiological cell responses and their potential as sources of specific antioxidant compounds [82,83,87].

In green microalgae, lutein, β-carotene, violaxanthin, antheraxanthin, zeaxanthin and neoxanthin have been identified as key components in the regulation of photosynthetic function and photoprotection. The reversible conversion of violaxanthin into antheraxanthin and zeaxanthin provides a rapid response to changes in irradiance, since the de-epoxidized xanthophylls facilitate thermal dissipation of excess excitation energy. Lutein has been demonstrated to contribute to the stability of the antenna and photoprotection. However, its accumulation is more closely coupled with active photosynthetic growth than with the marked stress-induced accumulation characteristic of some secondary carotenoids [89,90].

In the context of diatoms, fucoxanthin represents a significant pigment essential for fucoxanthin–chlorophyll protein complexes, thereby playing a central role in light harvesting processes. These organisms also utilize the diadinoxanthin–diatoxanthin cycle for accelerated photoprotection in conditions of excess light. Recent research conducted on *Phaeodactylum tricornutum* has elucidated several functions of enzymes implicated in xanthophyll cycling and fucoxanthin biosynthesis. This research has demonstrated a close relationship between light acclimation and the formation of this valuable carotenoid [91,92].

A distinction can be drawn between carotenoids associated mainly with photosynthetically active growth and those that are accumulated under environmental stress conditions. Primary carotenoids, including lutein, violaxanthin, zeaxanthin and fucoxanthin, have been found to be closely associated with photosynthetic complexes and photoprotection. The accumulation of secondary carotenoids, such as β-carotene and astaxanthin, has been observed in *Dunaliella salina* and *Haematococcus lacustris*, respectively, which accumulate them under adverse environmental conditions. This distinction is relevant for bioprocess design, as the environmental conditions that promote secondary carotenoid accumulation often occur at the expense of biomass productivity [85,93].

#### 3.1.2. Main Species and Carotenoid

The diversity of carotenoids found in microalgae is reflected by the wide range of species investigated for pigment production. The selection of a production platform depends on strain-specific pigment content, biomass productivity, responsiveness to cultivation conditions, ease of harvesting and downstream processing, and suitability for the intended application [65]. Thus, a small number of microalgal species have become established as promising sources of specific carotenoids, particularly β-carotene, astaxanthin, lutein, zeaxanthin, and fucoxanthin [84].

*Dunaliella salina* is the most well-established natural source of β-carotene. This halotolerant chlorophyte accumulates large amounts of β-carotene in plastid lipid globules under conditions of high irradiance, increased salinity, and nutrient limitation [94]. Related strains, particularly those from *Dunaliella bardawil*, have also been used to investigate production and recovery strategies for natural β-carotene. Recent studies have been conducted on the use of food-compatible extraction methods for carotenoid-rich and phytoene-rich *Dunaliella bardawil* biomass [95]. β-carotene is a natural orange pigment with high antioxidant activity and provitamin A properties. Its commercial relevance largely stems from its exceptional accumulation in *Dunaliella* species [96,97].

The production of astaxanthin is mainly associated with *Haematococcus lacustris*, which remains the principal natural source of this ketocarotenoid [98]. Under stress conditions, green vegetative cells differentiate into red, non-motile cells, with astaxanthin accumulating primarily as esterified forms within cytoplasmic lipid droplets. This response is promoted by high light or nutrient depletion stress and is accompanied by changes in photosynthesis, carbon allocation, and cell-wall properties [98]. *Chromochloris zofingiensis* is also being investigated as a potential alternative astaxanthin producer since it is able to grow rapidly and tolerate a variety of cultivation modes. However, its pigment profile and production strategy differ from those of *H. lacustris*, as was mentioned above [93,99].

Lutein is present at high concentrations in several chlorophytes, including *Chlorella sorokiniana*, *Chlorella vulgaris*, *Muriellopsis* spp., *Scenedesmus* spp., and *Desmodesmus* spp. [100]. In contrast to the stress-induced accumulation of astaxanthin and β-carotene, lutein production is generally associated with active cell growth, reflecting its essential role as part of the photosynthetic antenna complex [101]. Consequently, the most significant parameter for industrial production is not only the cellular lutein content but also volumetric productivity, which is influenced by the balance between biomass growth and pigment content. In this context, recent works have identified heterotrophic and mixotrophic cultivation, fed-batch operation, strain improvement, and metabolic engineering as key parameters for increasing lutein productivity in some microalgal strains [101,102].

Violaxanthin and neoxanthin are less established as commercial products from microalgae, but they are highly relevant to antioxidant protection and photoprotective responses in photosynthetic cells. Zeaxanthin and violaxanthin have been observed to participate in the xanthophyll-cycle response of green microalgae, while neoxanthin has been shown to contribute to the carotenoids associated with the photosynthetic apparatus [103,104,105]. Zeaxanthin, in combination with lutein, is of nutritional significance as a constituent of the macular xanthophylls [106]. It has been documented that these pigments were detected in various species of *Chlorella*, *Dunaliella*, and *Chlamydomonas*, in addition to other eukaryotic microalgae. The technological interest of these organisms is twofold. First, they represent valuable sources of bioactive compounds. Second, they serve as indicators of photosynthetic stress, acclimation, and light-use regulation [103,104,107,108].

Fucoxanthin is the principal carotenoid of interest in diatoms and several other marine microalgal lineages. *Phaeodactylum tricornutum* is the most extensively studied production platform because it combines high fucoxanthin content with available genetic resources and established cultivation methods [109]. Other relevant sources include *Pavlova* spp., *Isochrysis* spp., *Tisochrysis lutea*, *Odontella aurita*, and *Chaetoceros* spp. The productivity of fucoxanthin is influenced by irradiance, light spectrum, nitrogen supply, temperature, and cultivation mode [110,111].

#### 3.1.3. Antioxidant Capacity of Carotenoids

The antioxidant function of carotenoids is closely related to their molecular structure, particularly to the extended system of conjugated double bonds that characterizes this family of pigments. The polyene chain has been demonstrated to facilitate the deactivation of singlet oxygen by carotenoids, a process known as physical quenching. In the context of microalgae, these properties possess direct physiological relevance since carotenoids are located within or near thylakoid membranes and pigment–protein complexes. In these locations, they are exposed to oxidative pressure derived from photosynthetic activity [82]. It has been established that the antioxidant function of these systems is linked to the protection of photosystems, antenna complexes, and membrane lipids. This occurs under conditions in which the absorbed light energy exceeds its effective use in photochemistry and carbon fixation [88].

The presence and nature of terminal rings, hydroxyl or keto substituents, polarity, esterification states, and orientation within lipid bilayers have been demonstrated to influence the accessibility of the molecule to reactive species and its interaction with biological membranes. This structural diversity is of particular importance in the study of microalgae, given that their carotenoid profiles include highly hydrophobic carotenoids in addition to xanthophylls exhibiting divergent polarity and cellular function [86]. β-carotene stands out as a highly lipophilic hydrocarbon carotenoid with an efficient capacity to quench singlet oxygen [66,94]. In contrast, lutein and zeaxanthin are hydroxylated xanthophylls whose polar terminal groups interact with the hydrophilic regions of the membrane while the conjugated central chain remains associated with the hydrophobic core of the lipid bilayer [102]. The pathway also includes violaxanthin and antheraxanthin, which participate in the reversible xanthophyll cycle [104]. Further extending this structural diversity, astaxanthin is a ketocarotenoid containing hydroxyl and keto groups in both terminal rings, conferring greater polarity than β-carotene and facilitating its interaction with lipid membrane interfaces [93,98]. Meanwhile, fucoxanthin is a unique pigment containing an allenic bond, an epoxide group, hydroxyl substituents, and a conjugated carbonyl system functioning within fucoxanthin–chlorophyll protein complexes [109,112]. These distinct molecular configurations explain why carotenoids identified in eukaryotic microalgae differ markedly in their physiological functions, antioxidant relevance, and biotechnological potential, all of which are comprehensively summarized alongside their principal biological sources in Table 1.

Extensive experimental evidence from the literature highlights the distinct physiological behaviors and accumulation patterns of these representative carotenoids under stress conditions. In situations involving elevated irradiance, nutrient limitations, or salinity changes, the significance of these functions becomes pronounced [83]. For instance, in *Dunaliella* spp., β-carotene accumulates to high levels under stress to enhance protection against excessive irradiance [66,94]. However, its antioxidant behavior is strongly influenced by pigment concentration and oxygen partial pressure, as it may not act exclusively as a protective antioxidant under highly oxygenated systems [113,114]. Recent studies on *Dunaliella* by Sayegh et al. [96] and Bárcenas-Pérez et al. [97] evaluated β-carotene recovery using bacterial lipase-assisted methods and high-performance countercurrent chromatography, emphasizing the need for proper preservation to maintain its applied antioxidant properties. Regarding xanthophylls, experimental data show that lutein contributes to constitutive photoprotection [102], while zeaxanthin regulates non-photochemical energy dissipation to limit photooxidative damage under excess light [107]. Nutritionally, both xanthophylls have been shown to protect human retinal tissues against photooxidative damage [106]. In *Haematococcus lacustris*, astaxanthin accumulation accompanies the differentiation of green vegetative cells into red resting cells under severe environmental stress, where it is deposited in its esterified form within lipid droplets to ensure cell survival [93,98]. Finally, the high antioxidant potential of fucoxanthin-rich microalgal biomass has been validated in marine species like *Phaeodactylum tricornutum*, making this pigment highly suitable for integrated biorefinery strategies [92,112].

**Table 1:** Main carotenoids reported in eukaryotic microalgae: representative strains, physiological function, antioxidant relevance, and current applications.

Carotenoid	Representative Microalgal Strains	Current or Prospective Applications	References
β-Carotene (carotene)	*Dunaliella salina*; *Dunaliella* *bardawil*	Natural orange colorant, provitamin A ingredient, antioxidant-rich extracts, functional foods, nutraceuticals and cosmetic formulations.	[66,92,94,95]
Astaxanthin (ketoxanthophyll)	*Haematococcus lacustris*; *Chromochloris zofingiensis*	Aquaculture pigmentation, nutraceuticals, functional foods, cosmetics, and antioxidant formulations.	[14,19,20,40]
Lutein (xanthophyll)	*Chlorella sorokiniana*, *Chlorella vulgaris*, *Muriellopsis* spp., *Scenedesmus* spp., and *Desmodesmus* spp.	Nutritional formulations, eye-health ingredients, functional foods, natural yellow pigment, feed pigmentation, and cosmetic formulations.	[88,97,98,99,100,104]
Zeaxanthin (xanthophyll)	*Chlamydomonas reinhardtii*, *Chlorella* spp., and *Dunaliella* spp.	Nutritional formulations, functional ingredients, and engineered microalgal platforms for zeaxanthin-enriched biomass.	[7,24,29]
Neoxanthin (xanthophyll)	*Chlamydomonas reinhardtii*, *Chlorella* spp., and other chlorophytes	Carotenoid profiling and potential contribution to antioxidant-rich whole biomass or pigment extracts.	[80,84,103]
Fucoxanthin (oxygenated xanthophyll)	*Phaeodactylum tricornutum*, *Pavlova* spp., *Isochrysis* spp., *Tisochrysis lutea*, *Odontella aurita*, and *Chaetoceros* spp.	Functional foods, nutraceuticals, cosmetic formulations, and marine microalgal biorefineries.	[89,90,107,108,109,114]
Diadinoxanthin and diatoxanthin (diatom xanthophyll-cycle pigments)	*Phaeodactylum tricornutum*, *Chaetoceros* spp., and other diatoms	Useful indicators of photoprotective status in fucoxanthin-producing strains.	[12,13,37]

#### 3.1.4. Current and Prospective Applications of Carotenoids

The translation of carotenoid production from a laboratory context to industrial application is constrained by both biological and technological factors. For β-carotene and astaxanthin, pigment accumulation is commonly promoted under stress conditions that reduce biomass productivity, making two-stage cultivation strategies essential. In contrast, lutein production requires maximizing productivity while maintaining active cell growth. For fucoxanthin, major challenges include incomplete pathway characterization, moderate productivity in many strains, as well as the limited stability of the extracted pigment during processing and storage [90,92,93,112].

Downstream processing is also critical, as carotenoids are lipophilic and prone to degradation by oxygen, light, and temperature. Biomass pre-treatment, the extraction solvent, and formulation strategies strongly influence recovery efficiency and the suitability of the final product for food and nutraceutical applications. In this context, Morón-Ortiz et al. [95] evaluated food-grade green solvents for ultrasound-assisted carotenoid extraction from common and phytoene-rich *Dunaliella bardawil* biomass, identifying 2-methyltetrahydrofuran as a promising alternative to conventional solvents. These findings support the development of suitable carotenoid ingredients for food applications.

Bioaccessibility and stability after formulation are equally important, as carotenoids may undergo oxidation, isomerization, or poor dispersion in aqueous food matrices, reducing their functional value despite high pigment content. Consequently, encapsulation and emulsion-based delivery systems are being investigated to preserve pigment integrity and improve intestinal bioaccessibility. These strategies are particularly relevant for astaxanthin, β-carotene, and fucoxanthin, although they are broadly applicable to carotenoid-based functional ingredients [85,115].

**Figure 4:**
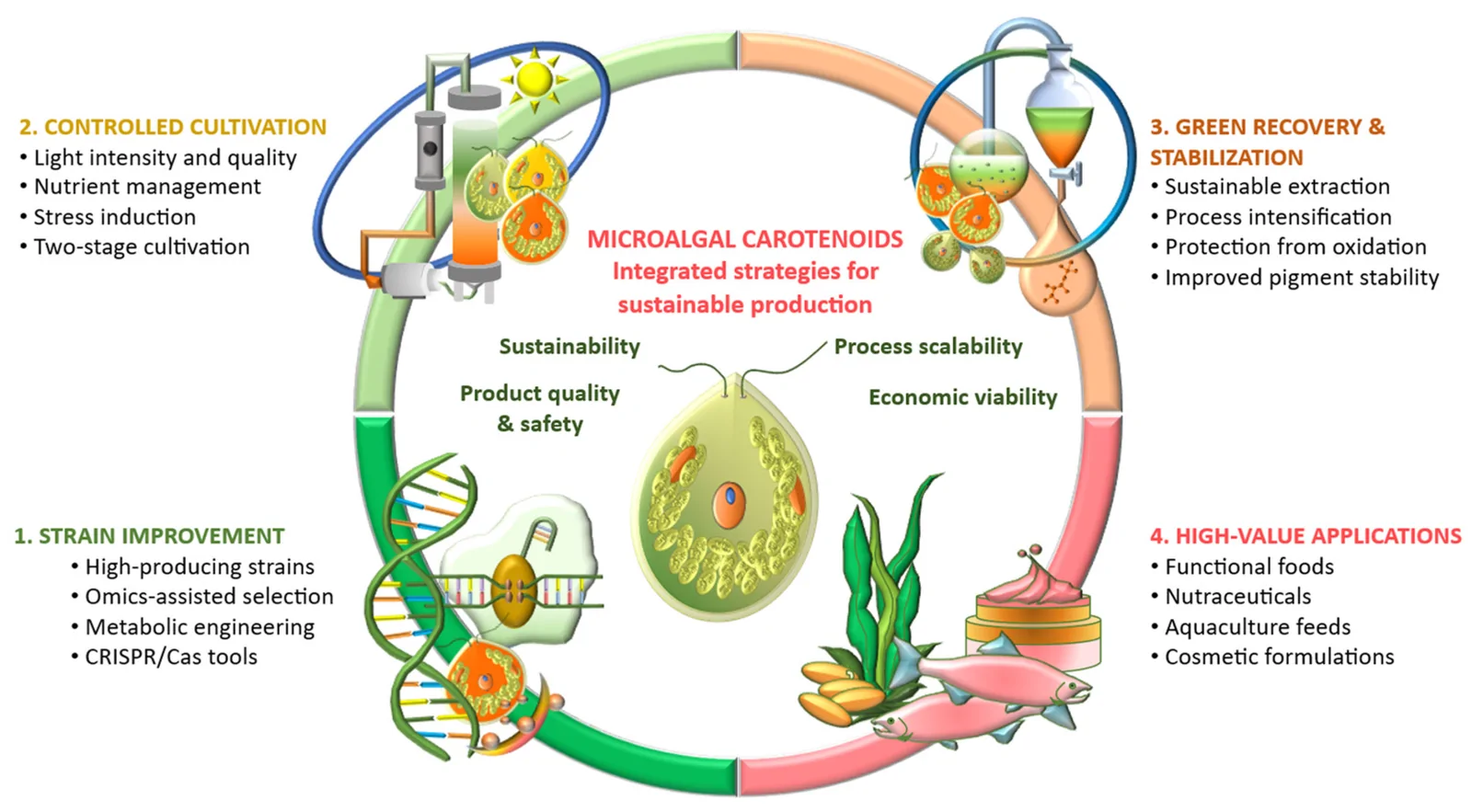
Integrated strategies for the production and application of microalgal carotenoids.

Recent advances in microalgal carotenoid production have been increasingly associated with the integration of optimized cultivation systems, molecular tools, and improved downstream processes to overcome the limitations associated with large-scale production (Figure 4). Controlled manipulation of irradiance, nutrient availability and stress exposure, including two-stage cultivation systems, enhances pigment accumulation while improving the balance between biomass production and carotenoid induction. Furthermore, targeted engineering of carotenoid biosynthetic pathways enables the redirection of metabolic flux towards compounds and the development of strains with tailored pigment profiles. In this regard, CRISPR-based engineering, pathway redesign, and omics-guided strain selection have emerged as promising approaches for improving carotenoid productivity and composition in microalgae [86,89]. However, the economic feasibility of large-scale production remains constrained by the trade-off between biomass productivity and pigment accumulation, as well as the costs associated with the harvesting, extraction, purification, and stabilization of oxidation-sensitive compounds (Figure 4). Thus, improving pigment productivity and downstream processing, while integrating the valorization of additional biomass fractions, may therefore contribute to enhancing process sustainability and the commercial competitiveness of carotenoid-based products [65].

As illustrated in Figure 4, the development of commercially viable microalgal carotenoids requires the integration of these advances across the entire production chain, from strain improvement and controlled cultivation to green recovery, stabilization and, ultimately, the development of high-value applications. An integrated biorefinery approach can further increase the value obtained from a single cultivation process by combining the valorization of lipids, proteins, and other biomass fractions, such as fatty acids, proteins, or vitamins [98,102]. Nevertheless, successful implementation of these stages requires simultaneous consideration of sustainability, product quality and safety, scalability, and economic viability. Thus, the commercial exploitation of microalgal carotenoids depends not only on appropriate strain selection but also on optimized extraction and formulation processes that preserve pigment integrity and antioxidant functionality [100]. Finally, the incorporation of carotenoids into high-value products for food, feed, nutraceutical, and cosmetic applications can increase their added value and contribute to the economic feasibility of the overall process [84,87,115]. Figure 4 provides an integrated framework linking upstream optimization, sustainable processing, biomass valorization and high-value applications, highlighting the main requirements for the production of microalgal carotenoids in sustainable and commercially viable platforms.

### 3.2. Fatty Acids

Lipids, whose content in microalgae ranges from 1% to 40% of dry weight under nutrient-replete culture conditions, play essential roles in membrane organization, cell architecture, and signaling. They also serve as carbon and energy reserves under stress conditions [116]. Under these conditions, lipid content may increase up to 60% of the dry weight and is often accompanied by the accumulation of other storage compounds, such as starch [117,118]. Among non-polar lipids, PUFAs have attracted particular interest due to their role in cellular responses to oxidative stress. Rather than acting as direct radical scavengers, PUFAs contribute to antioxidant defense by maintaining membrane integrity, supporting plastid stabilization, and modulating cellular responses to oxidative stress [119].

Fatty acid profiles, particularly PUFA composition, are characteristic of different microalgal taxa and are widely used in their systematic classification [22]. In addition, abiotic factors such as salinity, irradiance, temperature, or nutrient availability influence FA composition, demonstrating the metabolic plasticity of microalgae to environmental conditions [120,121]. This adaptative response has been extensively exploited to optimize the large-scale production of bioactive FAs for food and pharmaceutical applications.

#### 3.2.1. Metabolic Importance of Fatty Acids

Fatty acids (FAs) are essential structural components required for the biosynthesis of membrane and storage lipids. Despite variations among microalgal taxa, the initial steps of de novo FA biosynthesis are highly conserved. Within plastids, acetyl-CoA carboxylase catalyzes the conversion of acetyl-CoA into malonyl-CoA, which is subsequently transferred to the fatty acid synthase (FAS) complex via an acyl carrier protein. FAS catalyzes successive cycles of condensation, initial reduction, dehydration, and final reduction, resulting in the synthesis of the backbone up to 16 carbons (palmitic acid, C16:0) or 18 carbons (stearic acid, C18:0), the primary precursors of longer FAs [122].

Subsequently, very-long-chain PUFAs are synthesized predominantly in the endoplasmic reticulum through the coordinated action of elongases, which extend the carbon chain, and desaturases, which increase the degree of unsaturation [123]. Based on the location of the first double bond from the terminal methyl of the chain, PUFAs are classified as ω3 or ω6 FAs. Among the most commercially relevant are γ-linolenic acid (GLA, C18:3, ω6), α-linolenic acid (ALA, C18:3, ω3), arachidonic acid (ARA, C20:4, ω6), eicosapentaenoic acid (EPA, C20:5, ω3) and docosahexaenoic acid (DHA, C22:6, ω3). Table 2 summarizes the main PUFAs containing three or more unsaturations identified across microalgal taxonomic groups.

EPA and DHA are the PUFAs with the greatest biotechnological potential. Haptophytes and dinoflagellates are the main producers of DHA, whereas diatoms are the primary producers of EPA [119].

FAs, including PUFAs, confer hydrophobicity to diacylglycerol molecules, which consist of two fatty acid residues attached to a glycerol backbone. This backbone can be linked to a polar glycosylated derivative, forming glycolipids, or to a phosphate group linked to a sugar or amine group, forming phospholipids. These polar lipids are the major components of cellular and thylakoid membranes. The amphipathic nature contributes to membrane fluidity and organization [116,121]. Unsaturated FAs are enriched in plastidial structures [131], and their abundance decreases drastically under stress conditions due to membrane remodeling and degradation. The characteristic distribution of FAs within thylakoid membranes suggests that they play essential roles not only in membrane delimitation but also in maintaining redox homeostasis and photosynthetic performance [132].

Regarding storage lipids, the attachment of three identical or different FA chains into a glycerol backbone forms triacylglycerols (TAGs), which constitute the major carbon reserves of the cell under unfavorable conditions [133]. TAGs are synthesized either de novo, a process reported to occur in both plastids and the endoplasmic reticulum [134], or through recycling mechanisms, in which membrane turnover supplies the FAs required for TAG production [135]. TAGs are stored in lipid droplets, providing an energy reserve for cell maintenance. When environmental conditions become favorable, carbon remobilization takes place, and FAs are released through the action of lipases [136].

To a lesser extent, oxygenated FA derivatives, known as oxylipins, are produced under oxidative stress [137]. Oxylipins are generated through either enzymatic or non-enzymatic oxidation mechanisms. Lipoxygenases and dioxygenases catalyze the former, whereas free radicals mediate the latter. These reactions generate a wide range of oxidized metabolites, including hydroxy-fatty acids, short-chain polyunsaturated aldehydes, or signaling molecules such as prostaglandins or protectines, which mediate the cellular stress response [138]. In diatoms, oxylipins act as signaling molecules, triggering programmed cell death through intracellular Ca^2+^ and by increasing NO production [139].

#### 3.2.2. Main Species and Fatty Acids

**Figure 5:**
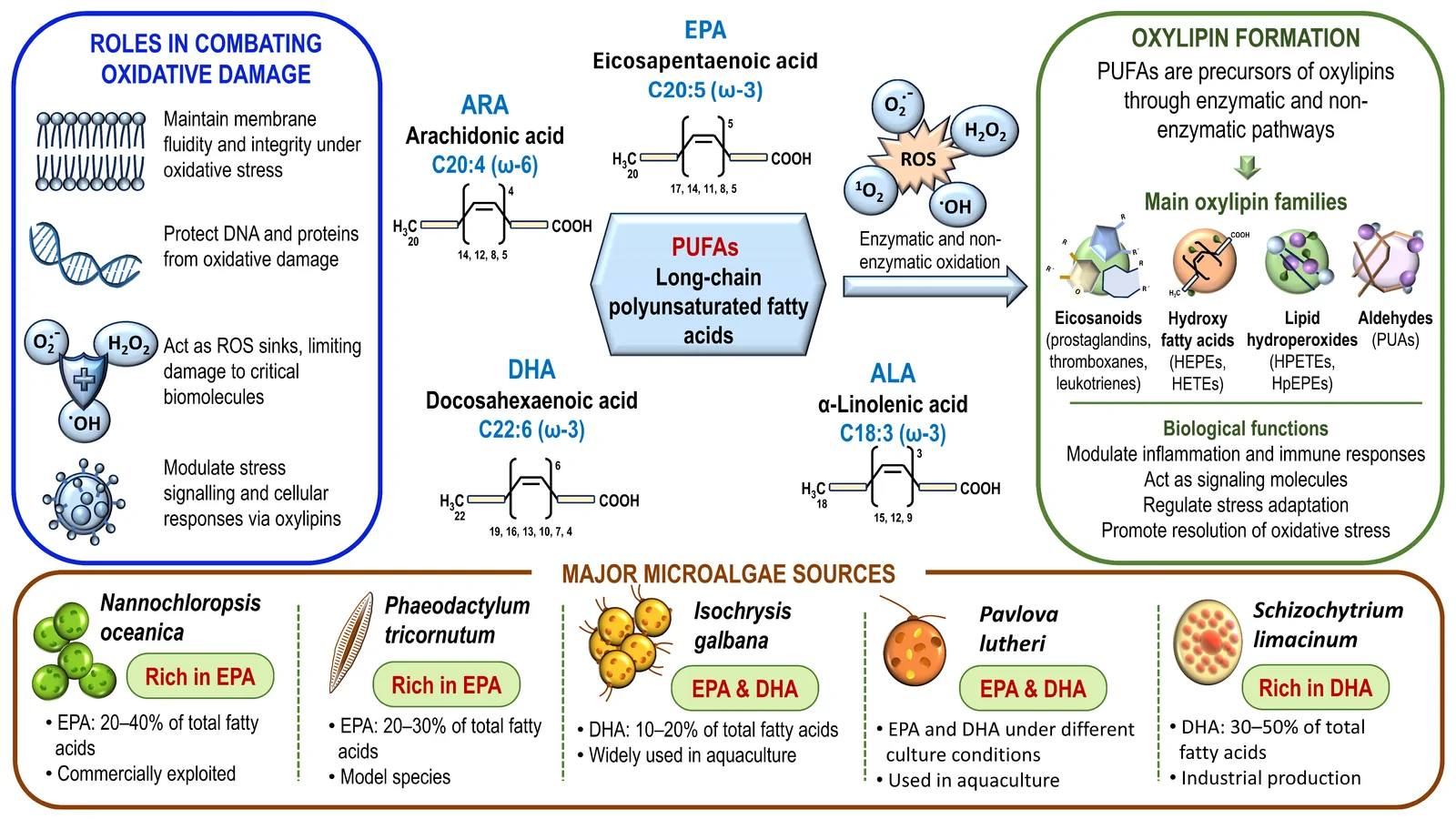
Main PUFAs and derivatives with biotechnological potential and major microalgal sources.

Several groups of microalgae are enriched in very-long-chain PUFAs. EPA is generally obtained from diatoms, and DHA is obtained from dinoflagellates and haptophytes [119]. Figure 5 summarizes the main microalgal species associated with their production.

*Schizochytrium* sp. is one of the most studied microorganisms for PUFA production due to its exceptional capacity to accumulate DHA. Its commercial use is well established in the nutraceutical industry. Several cultivation strategies have been developed to enhance DHA accumulation, including the optimization of large-scale bioreactors, achieving a DHA content of up to 30.5% of the biomass [140]. The addition of phytohormones and antioxidants to the culture medium has also increased DHA accumulation [141]. Metabolic engineering has likewise been applied to *Schyzochytrium* to enhance DHA biosynthesis. Wang et al., 2019 achieved a 1.4-fold increase in DHA levels by deactivating the inhibition of acetyl-CoA carboxylase caused by the pool of C16-CoA in the cell and overexpressing an *ELO3* gene from *Mortierella alpina* [142]. Furthermore, overexpression of acetyl-CoA synthetase, which increases carbon flux toward the FAS pathway, resulted in an 11.3% increase in total lipid content [143].

*Phaeodactylum tricornutum* is a marine diatom that accumulates high levels of EPA in its polar lipids. Increasing urea and CO_2_ concentrations enhanced EPA content by 38.6% and 73%, respectively, compared to the control culture [144]. Process optimization in pilot-scale batch cultures using kinetic modeling increased biomass productivity by 50% and EPA production by 20% [145]. Nitrogen deprivation, one of the most widely applied strategies for lipid induction in microalgae, increased total lipid content but reduced the PUFA/saturated FA ratio in *Phaeodactylum tricornutum*. Molecular engineering has also been extensively applied to this species, for which genetic engineering protocols are well established. Co-expression of a Δ5 elongase from *Ostreococcus tauri* and a glucose transporter from *Physcomitrium patens* enhanced EPA and DHA content [146], whereas overexpression of an endogenous Δ6 desaturase increased EPA levels by nearly 48% [147].

*Nannochloropsis*, another genus of marine microalgae, is also used for EPA production, which can account for up to 30% of the total cellular lipids. It is already commercially available as whole biomass for dietary supplementation [148]. Different cultivation strategies can modify the distribution of EPA among lipid classes. Sá et al., 2020 reported increased EPA accumulation in the TAG fraction under nitrogen depletion and low irradiance, whereas low light or low temperatures promoted its accumulation in the phospholipid fraction [149]. Regarding genetic engineering, the simultaneous overexpression of a Δ5 elongase and ω3 desaturases in *Nannochloropsis oceanica* boosted EPA levels in both replete and N-starved culture conditions [150].

Unlike *Nannochloropsis*, *Isochrysis galbana* still lacks robust genetic engineering platforms; therefore, PUFA enhancement mainly relies on strain selection and cultivation optimization. The use of dough waste, along with optimized combinations of carbon sources (CO_2_, glucose, and amino acids), improved both biomass productivity and DHA content [151,152]. *Pavlova lutheri* is another haptophyte of high interest due to its capacity to synthesize both EPA and DHA. Although it has similar limitations regarding routine genetic engineering, random UV mutagenesis has successfully increased the total PUFA content by 30% relative to the parental strain [153].

#### 3.2.3. Antioxidant Capacity of Fatty Acids

The antioxidant role of lipids, particularly polyunsaturated fatty acids (PUFAs), operates through indirect and structural mechanisms rather than classical radical scavenging. The presence of multiple double bonds makes PUFAs highly susceptible to peroxidation by ROS. However, they are not considered classical antioxidants in microalgae, as their oxidation is not primarily intended to prevent oxidative damage to specific cellular targets [154]. Instead, PUFA oxidation generates bioactive metabolites that activate physiological responses under adverse environmental conditions, thereby contributing indirectly to protective mechanisms against oxidative stress [138]. Structurally, under adverse conditions, such as high temperatures, the degree of FA unsaturation in thylakoid membranes plays a major role in maintaining the structural and functional integrity of photosynthetic complexes. High PUFA levels preserve photosystem stability and photosynthetic electron transport efficiency, while their partial saturation counteracts the increase in membrane fluidity caused by heat stress to reduce proton leakage, helping maintain the electrochemical gradient and photosynthetic efficiency [155].

The primary representative lipid components involved in these defense pathways include thylakoid-bound ω-3 PUFAs and their downstream enzymatic or non-enzymatic oxidation products, collectively known as oxylipins. Within microalgae and related photosynthetic organisms, these oxidized fatty acid derivatives encompass a diverse range of molecules, including C18 and C20 oxylipins, as well as volatile compounds such as polyunsaturated aldehydes [139].

Substantial experimental evidence supports the dual structural and signaling roles of these lipid compounds under stress conditions. For instance, *Arabidopsis* mutants deficient in chloroplast ω-3 PUFAs exhibited greater photoinhibition and lower thermotolerance than both wild-type and overexpressing lines [132]. Moreover, thylakoidal PUFAs in *Phaeodactylum tricornutum* undergo partial saturation under high temperatures to reduce proton leakage during photosynthetic electron transport [155]. Linares-Maurizi et al., 2024 reported that H_2_O_2_ supplementation directly increased oxylipin production in microalgal cultures, confirming that oxylipin synthesis is upregulated under oxidative stress due to PUFA peroxidation [156]. Furthermore, experimental data reveal a dual ecological and defensive function for these molecules; in microalgae, volatile polyunsaturated aldehydes attract copepods or gastropods while simultaneously reducing hatching success to prevent predation, whereas in the red alga *Chondrus crispus*, the production of C18 and C20 oxylipins was significantly induced by pathogen exposure, demonstrating their active role in immune responses [157].

#### 3.2.4. Current and Prospective Applications of Fatty Acids

As ω3 and ω6 FAs are essential in human and animal nutrition, their commercial production is well established, with plant oils and fish oil as their primary sources. Microalgae are considered an emerging source of these compounds, although their large-scale production remains less developed, with DHA representing the commercial exception [117]. Indeed, the high EPA and DHA content of fish oil originates largely from microalgal consumption. Ebm et al., 2021 showed that PUFA accumulation in freshwater fish tissues reflected a greater contribution from microalgal intake than from other dietary sources, such as leaves [158]. Consequently, *Schyzochytrium* sp. is widely used in aquaculture to increase the nutritional value of species, including Atlantic salmon, rainbow trout, or broiler chicken [131].

Their antioxidant and anti-inflammatory properties have also supported their use as nutritional supplements. EPA and DHA are critical for maintaining the brain and retinal membranes, contributing to their development and integrity. In addition, in patients undergoing chemotherapy, EPA supplementation has been proven to modulate pro-inflammatory cytokines, reducing treatment-associated muscle and weight loss [159].

Beyond nutritional applications, microalgal PUFAs have also been associated with the prevention of cardiovascular diseases [160]. As discussed above, ω3 PUFAs are essential for adequate brain function, and their intake has also been associated with improved symptoms of depression in teenagers [161]. Mola et al., 2025 further demonstrated that myeloperoxidase activity associated with HDL oxidation is inversely correlated with higher ω3 and ω6 PUFA intake [162]. Moreover, they are increasingly being explored as cosmetic ingredients because of their contribution to skin barrier function, moisturizing properties, and potential anti-aging applications [163].

Oxylipins have attracted increasing biomedical interest because of their cytotoxic activity. In mice studies, treatment of TNBS-induced colitis with oxylipins derived from the microalga *Chlamydomonas debaryana* reduced pro-inflammatory cytokine levels and restored the colon morphology. Furthermore, the combination of hydroxyl-fatty acid 13-HOTE with the anticancer drug 5-fluorouracil produced a synergistic cytotoxic effect on HT-29 colon adenocarcinoma cells [164,165]. Likewise, in vitro cultures of the breast cancer cell line BT20 showed an apoptosis rate of 89.2% following treatment with the EPA-enriched oxylipin fraction from the diatom *Cocconeis scutellum* [139], which demonstrates the promising potential of microalgal PUFA derivatives as adjuncts in cancer therapy.

Overall, microalgal PUFAs are high-value compounds with promising applications in the nutraceutical, pharmaceutical, and aquaculture sectors. Optimization of large-scale production through appropriate growth and metabolite induction strategies will be essential to ensure their economically viable commercialization.

### 3.3. Liposoluble Vitamins

Microalgae are exceptional sustainable sources of fat-soluble vitamins. They synthesize these compounds naturally as structural components of their photosynthetic machinery or as metabolic defenses against environmental stress. Due to their high physiological plasticity, the accumulation of these vitamins can be significantly upregulated through environmental stress and cultivation engineering, offering a scalable, vegan alternative to animal-derived nutrients for the food, cosmetic, and nutraceutical industries.

#### 3.3.1. Metabolic Importance of Liposoluble Vitamins

Liposoluble vitamins, including vitamins A, D, E, and K and their metabolic precursors, play essential roles in microalgal physiology. These compounds contribute to membrane stability, photoprotection, photosynthetic electron transport, and maintenance of cellular redox homeostasis [166,167,168,169]. Their biosynthesis is tightly associated with oxygenic photosynthesis and influenced by the oxidative environment generated under high irradiance. Excess light promotes the formation of ROS, particularly singlet oxygen and lipid peroxyl radicals within thylakoid membranes, increasing the requirement for molecules such as tocopherols and provitamin A [169,170].

Vitamin A, like other apocarotenoids, is biosynthesized from provitamin A carotenoids, particularly β-carotene. Plants, fungi, and microalgae cannot synthesize retinol directly. Instead, they produce provitamin A carotenoids that the human body must ingest and break down using intestinal enzymes. Provitamin A is essential in microalgae cells, as it is a component of the photosynthetic apparatus, contributing to pigment–protein complex stability, photosystem function, and singlet oxygen quenching under excess light [66,169].

Vitamin E (tocopherol) is located in thylakoid membranes, where it preserves membrane integrity by limiting lipid peroxidation and maintaining photosynthetic function under oxidative conditions, thereby contributing to the maintenance of membrane integrity [171,172,173].

Vitamin K_1_ (phylloquinone) is an essential redox cofactor in photosystem I (PS I), serving as the A1 electron acceptor in the photosynthetic electron transport chain. It has also been associated with acclimation to environmental conditions in microalgae and cyanobacteria [174].

Vitamin D, on the other hand, is rarely synthesized directly by microalgae. Instead, several species accumulate provitamin sterols, such as ergosterol and 7-dehydrocholesterol, which are photochemically converted into vitamin D2 and vitamin D3, respectively, under UV radiation. This process represents an indirect photoprotective response to high irradiance [166].

#### 3.3.2. Main Species and Liposoluble Vitamins

Liposoluble vitamins and their precursors are unevenly distributed among microalgae, with their accumulation depending on both taxonomic groups and cultivation conditions [170,172]. Chlorophytes, diatoms, haptophytes, and eustigmatophytes include species capable of producing lipophilic vitamins or their metabolic precursors with nutritional and industrial value [169,172,175].

Environmental factors, particularly irradiance, salinity, and nutrient availability, strongly regulate vitamin biosynthesis. Consequently, cultivation under abiotic stress usually promotes the accumulation of vitamins and has been widely exploited to enhance vitamin productivity [176].

Among provitamin A-producing microalgae, *Dunaliella salina* remains the most representative species. Under standard cultivation, β-carotene contents are typically below 1 mg g^−1^ DW, whereas high irradiance combined with nitrate limitation can increase concentrations to approximately 140 mg g^−1^ DW [45]. Intermediate provitamin A contents have been reported for *Chlorella vulgaris*, *Chaetoceros* spp., *Tetraselmis suecica*, *Porphyridium* spp., and *Phaeodactylum tricornutum*, while *Nannochloropsis* spp. generally exhibit comparatively lower concentrations [177].

Vitamin E is predominantly found as α-tocopherol in microalgae, although γ-tocopherol represents the major isoform in *Tetraselmis suecica* and *Chaetoceros* spp. [172,178,179]. Tocopherol accumulation also depends on cultivation conditions. *Tetraselmis suecica* exhibits the highest reported concentrations (6.32 to 19.8 mg g^−1^ DW) under optimized cultivation [180,181,182], whereas *Dunaliella salina* shows a 1.5-fold increase after UV-B exposure [183].

Compared with information on provitamin A and vitamin E, information on vitamin K_1_ in microalgae is considerably more limited. Available studies indicate that phylloquinone is generally present at low concentrations and that its accumulation has not been consistently associated with the stress response. The highest reported content corresponds to *Skeletonema costatum*, *Isochrysis galbana*, and *Pavlova lutheri*, whereas *Tetraselmis suecica* contains substantially lower levels [74].

Vitamin D metabolism differs from that of other fat-soluble vitamins. Rather than accumulating constitutively, most microalgae synthesize sterol precursors, such as ergosterol and 7-dehydrocholesterol, which undergo photochemical conversion into vitamins D2 and D3 following UV-B exposure [166,183]. While basal vitamin D2 and D3 concentrations remain extremely low under standard cultivation, UV-B treatment enhances their accumulation, particularly in *Dunaliella salina* and *Nannochloropsis* spp. [183], highlighting the critical role of UV irradiation in vitamin D biosynthesis.

#### 3.3.3. Antioxidant Capacity of Liposoluble Vitamins

Fat-soluble vitamins and their precursors constitute the principal lipophilic antioxidant system in microalgae and are interesting for application in many fields, as shown in Table 3. Owing to their location within cellular and thylakoid membranes, they protect membrane lipids, preserve photosynthetic function, and contribute to redox homeostasis under adverse environmental conditions [166,167,168,169]. Direct antioxidant mechanisms include singlet oxygen quenching within antenna complexes and reaction centers, as well as the scavenging of lipid peroxyl radicals, which effectively limits photooxidative damage and prevents destruction of chloroplast structures under high irradiance [184]. Additionally, they protect thylakoid membranes by scavenging lipid peroxyl radicals, preventing damage to chloroplast structures [185]. Indirectly, these lipophilic molecules contribute to redox regulation by ensuring efficient electron transport through the photosynthetic chain—thereby preventing over-reduction and limiting upstream ROS generation—or by modulating membrane organization and driving physiological and transcriptomic responses involved in stress acclimation [176,186].

The primary representative compounds within this lipophilic network encompass four major vitamin classes: provitamin A (predominantly represented as β-carotene, located within the light-harvesting complexes and reaction centers) [187], vitamin E (mainly α-tocopherol, along with minor concentrations of tocotrienols) [172], vitamin K1 (phylloquinone, which functions as the A_1_ electron acceptor in PSI) [188,189], and vitamins D_2_ and D_3_ (synthesized through the UV-induced photoconversion of ergosterol and 7-dehydrocholesterol, respectively) [166].

Extensive experimental evidence highlights the accumulation and functional dynamics of these fat-soluble vitamins under environmental constraints. Because provitamin A accumulation increases under high light, salinity stress, or nutrient limitation, its protective capacity is closely linked to the acclimation of the photosynthetic apparatus to varying environmental conditions [66,170,190]. Similarly, experimental data confirm that vitamin E (α-tocopherol) accumulation is enhanced under identical environmental stress conditions, such as high irradiance, salinity, or nutrient limitation [185,191,192]. Regarding vitamin K1, the literature establishes its structural role in maintaining efficient electron flow through the photosynthetic apparatus to limit ROS generation [188,189,193]. Finally, UV-induced vitamin D biosynthesis has been experimentally linked to physiological and transcriptomic responses that enhance overall cellular tolerance to environmental stress [176,186].

**Table 3:** Liposoluble vitamins produced by microalgae and biotechnological applications.

Liposoluble Vitamin/Precursor	Main Microalgal Sources	Potential Application	References
Provitamin A (β-carotene)	*Dunaliella salina*, *Tetraselmis suecica*, *Chlorella vulgaris*, *Isochrysis galbana*, *Chaetoceros* spp., and *Nannochloropsis* spp.	Provitamin A from a natural source for food and feed; nutraceutical antioxidants; natural colorant; biofortification. Commercial production is established especially for the biomass of *Dunaliella*	[66,169,170,184,187]
Vitamin E (tocopherols)	*Nannochloropsis oculata*, *Tetraselmis suecica*, *Chaetoceros calcitrans*, *Porphyridium purpureum*, and *Chlorella* spp.	Functional foods; antioxidant ingredients; oxidative stabilization of oils; aquaculture feed enrichment; cosmetic formulations.	[171,172,173,178,194,195]
Vitamin E (tocotrienols)	*Scenedesmus* sp. (reported occurrence); evidence in microalgae remains limited compared with tocopherols	Investigated for nutraceutical and metabolic health applications; evidence of microalgal production remains exploratory.	[172,194,196]
Vitamin K_1_ (phylloquinone)	*Chlorella vulgaris*, *Tetraselmis suecica*, chlorophytes and diatoms with photosystem I	Nutritional supplementation and development of vitamin K-enriched microalgal biomass; proposed functional food ingredient.	[174,189,193]
Vitamin D_2_ (UV-derived from sterol precursors)	*Pavlova lutheri*, *Tetraselmis suecica*, *Dunaliella salina*, *Skeletonema costatum*, and *Emiliania huxleyi*	Functional ingredients.	[166,176,186]
Vitamin D_3_ (UV-induced production and/or sterol conversion)	*Dunaliella salina*, *Nannochloropsis limnetica*, *Nannochloropsis oceanica*, and *Emiliania huxleyi*	Emerging route for sustainable vitamin D production and fortified biomass generation.	[166,176,183,186]
Vitamin D precursors (ergosterol, 7-dehydrocholesterol, and related sterols)	*Emiliania huxleyi*, *Dunaliella tertiolecta*, *Chlamydomonas reinhardtii*, *Chlorella vulgaris*, and *Tetraselmis suecica*	Biotechnological platforms for controlled photoconversion and vitamin D biofortification.	[166,197,198]

#### 3.3.4. Current and Prospective Applications of Liposoluble Vitamins

Lipid-soluble vitamins are essential micronutrients involved in vision, immune regulation, bone metabolism, blood coagulation, and antioxidant defense, making them valuable ingredients for food, pharmaceutical, and feed applications [172,199].

Provitamin A, derived mainly from β-carotene, is extensively incorporated into functional foods, dietary supplements, pharmaceuticals, and animal feed due to its antioxidant properties and its role in visual health, immune function, and photoprotection [200,201]. Likewise, vitamin E is extensively applied in nutraceuticals and cosmetic formulations because it protects biological membranes from lipid peroxidation and contributes to preventing cardiovascular, metabolic, and immune health issues [172]. Vitamins D and K are also essential high-value compounds due to their roles in calcium homeostasis, bone metabolism, vascular health, and blood coagulation [202].

As humans are unable to synthesize these vitamins de novo, sustainable dietary sources remain of considerable interest. In this context, microalgae are a promising alternative owing to their high productivity, metabolic flexibility, and ability to yield vitamins and other high-value metabolites simultaneously [167]. Species such as *Chlorella*, *Spirulina*, *Dunaliella*, *Haematococcus*, *Emiliania*, *Skeletonema*, and *Nannochloropsis* naturally accumulate liposoluble vitamins or their precursors, highlighting their potential as multifunctional biofactories [203]. Among them, *Dunaliella salina* is the primary commercial source of β-carotene, while *Nannochloropsis oceanica* provides a unique combination of proteins, EPA, and vitamin K, highlighting its potential as a multifunctional nutritional ingredient [66]. Since cultivation conditions strongly influence vitamin concentrations, optimizing irradiance, temperature, and salinity remains essential to maximize biomass productivity and enhance process efficiency [66,204].

Future developments should focus on improving biomass productivity, vitamin recovery, and process economics through optimized cultivation, metabolic engineering, and sustainable downstream technologies [170,172]. Green extraction approaches, including supercritical CO_2_ and high-pressure processes, together with integrated biorefinery strategies, are expected to enhance the economic viability of microalgal vitamin production for food, feed, cosmetic, and pharmaceutical applications [172,204,205].

### 3.4. Hydrophilic Vitamins

In addition to their lipophilic counterparts, microalgae are rich sources of water-soluble vitamins that operate primarily within the aqueous phases of the cytoplasm, mitochondria, and chloroplast stroma. Given their high bioavailability and the capacity of microalgal biomass to accumulate them under optimized cultivation conditions, these water-soluble vitamins represent a sustainable and highly valuable matrix for functional food development and target human nutrition.

#### 3.4.1. Metabolic Importance of Hydrophilic Vitamins

Hydrophilic vitamins constitute a functionally diverse group of bioactive metabolites that are crucial for microalgal growth, metabolism, and stress adaptation. In addition to acting as essential cofactors in metabolic pathways, these compounds contribute significantly to antioxidant defense by mitigating oxidative damage and maintaining cellular redox balance in microalgae. Some of the most relevant hydrophilic vitamins are ascorbic acid (vitamin C) and several B-complex vitamins, such as thiamine (vitamin B1), riboflavin (vitamin B2), niacin (vitamin B3), pyridoxine (vitamin B6), folate (vitamin B9), and cobalamin (vitamin B12) [74,206]. These molecules are involved in processes related to photosynthesis, electron transport chains, energy metabolism, and the maintenance of cellular redox homeostasis, sustaining cellular homeostasis and enhancing microalgal resilience to environmental stressors [30,207,208].

When ROS levels exceed the scavenging capacity of antioxidant defense systems in microalgae, oxidative stress ensues, producing detrimental alterations in proteins, lipids, nucleic acids, or photosynthetic pigments, thereby compromising cellular integrity and metabolic performance [209,210]. To counteract these effects, microalgae have developed a complex network of antioxidant mechanisms to maintain cellular homeostasis, in which hydrophilic vitamins play a crucial role, contributing to ROS detoxification and cellular protection. Among these compounds, ascorbic acid (vitamin C) is regarded as one of the most important antioxidants in photosynthetic organisms. Its central role in the ascorbate–glutathione cycle, considered one of the major pathways involved in cellular ROS detoxification, enables the efficient scavenging of these molecules and the regeneration of other antioxidant molecules [211,212]. Furthermore, it contributes to the photoprotection of photosystems I and II by mitigating photooxidative damage and supporting the maintenance of photosynthetic efficiency under stress conditions [211].

In addition to ascorbic acid, several B-complex vitamins play a crucial role in the regulation of cellular redox metabolism and antioxidant defense. Riboflavin (vitamin B2) acts as a precursor to flavin mononucleotide (FMN) and flavin adenine dinucleotide (FAD), essential cofactors for numerous oxidoreductases involved in electron transport, energy metabolism, and cellular respiration [74]. Pyridoxine (vitamin B6) contributes to oxidative stress mitigation through its capacity to quench singlet oxygen and reduce lipid peroxidation processes [213]. Likewise, folate (vitamin B9) and cobalamin (vitamin B12) are involved in carbon metabolism and amino acid biosynthesis, thereby supporting glutathione production and the maintenance of intracellular redox homeostasis [214].

Because of their diverse physiological functions and recognized health benefits, vitamins have attracted increasing interest in recent years as sustainable bioactive compounds for food, pharmaceutical, and nutraceutical applications [215,216,217]. In this context, microalgae have emerged as a promising and alternative source of vitamins, particularly cobalamin (vitamin B12) for vegetarian and vegan diets due to its limited availability in plant-derived foods [206].

#### 3.4.2. Main Species and Hydrophilic Vitamins

The biosynthesis and accumulation of hydrophilic vitamins of biotechnological interest have been reported in a wide range of marine and freshwater microalgae, as shown in Table 4. Among the most investigated species are *Chlorella vulgaris*, *Arthrospira plantensis*, *Dunaliella salina*, *Tetraselmis suecica*, and *Nannochloropsis gaditana*, which have attracted considerable interest owing to their capacity to produce valuable vitamins and other bioactive compounds [74].

Among these species, *Chlorella vulgaris* is one of the most widely exploited microalgae for nutritional and biotechnological applications due to its high nutritional value and its ability to accumulate high content of B-complex vitamins, particularly riboflavin (vitamin B2), folate (B9), and cobalamin (vitamin B12) [218,219]. In addition, this species contains significant amounts of ascorbic acid (vitamin C), whose accumulation can be further enhanced under environmental stress conditions, including high irradiance and nitrogen limitation [30,220]. These characteristics positioned *Chlorella vulgaris* as a promising platform for the sustainable production of vitamin-enriched biomass.

**Table 4:** Hydrophilic vitamins produced by microalgae, their antioxidant functions, and potential biotechnological applications.

Hydrophilic Vitamin	Main Microalgal Sources	Potential Applications	References
Vitamin C (ascorbic acid)	*Chlorella vulgaris*, *Dunaliella salina*, and *Tetraselmis suecica*	Nutraceuticals, cosmetics, functional foods, and antioxidant supplements	[30,211,212,220]
Vitamin B1 (thiamine)	*Chlorella* spp. and *Nannochloropsis* spp.	Functional foods and nutritional supplementation	[74,208,215]
Vitamin B2 (riboflavin)	*Chlorella vulgaris* and *Arthrospira platensis*	Nutraceuticals, food fortification, and pharmaceutical formulations	[30,74,216]
Vitamin B3 (niacin)	*Chlorella* spp. and *Tetraselmis* spp.	Functional foods and dietary supplements	[74,208]
Vitamin B6 (piridoxine)	*Chlorella vulgaris* and *Tetraselmis suecica*	Antioxidant formulations and nutraceuticals	[213,220]
Vitamin B9 (folates)	*Nannochloropsis gaditana* and *Chlorella vulgaris*	Food fortification and dietary supplementation	[30,214]
Vitamin B12 (cobalamin)	*Chlorella vulgaris*, *Arthrospira* spp., and cyanobacteria	Vegan supplementation, functional foods, and nutraceuticals	[206,210,218,219]

Species belonging to the genus *Arthrospira* are also of considerable commercial importance and are widely marketed as dietary supplements due to their high protein content and abundance of bioactive compounds [216,221]. However, numerous studies have demonstrated that a substantial proportion of the vitamin B12 detected in cyanobacteria of the genus *Arthrospira* corresponds to pseudocobalamin, a cobalamin analog with limited bioavailability in humans [206,218,219]. Despite this limitation, *Arthrospira* remains highly attractive for nutraceutical applications because of its favorable nutritional profile, antioxidant properties, and established large-scale cultivation systems [210].

Other microalgal species, such as *Dunaliella salina* and *Tetraselmis suecica*, have also demonstrated a remarkable capacity to accumulate hydrophilic vitamins and other antioxidant metabolites when exposed to environmental stressors such as high light irradiance or osmotic stress [30,220]. The biosynthesis and intracellular accumulation of these compounds are strongly influenced by cultivation parameters, including high light intensity, temperature, salinity, and nutrient availability [169,209,220]. As a result, the application of controlled stress has emerged as a widely adopted cultivation strategy to enhance the accumulation of high-value metabolites [220,222].

#### 3.4.3. Antioxidant Capacity of Hydrophilic Vitamins

Hydrophilic vitamins derived from microalgae contribute significantly to the antioxidant potential of microalgal biomass through both direct radical-scavenging activity and indirect modulation of cellular redox processes [30,207]. These compounds also constitute an integral component of the non-enzymatic antioxidant defense network (Figure 3) that enables microalgae to cope with adverse environmental conditions. Direct mechanisms of action involve electron donation to neutralize various ROS, as well as the specific ability to quench singlet oxygen and prevent lipid peroxidation. These direct pathways are essential to protect photosystems against photooxidative damage and preserve the integrity of cellular membranes, pigments, and proteins from photosynthetic byproducts [211,212]. Indirectly, these compounds maintain intracellular redox homeostasis by serving as central components in the ascorbate–glutathione cycle or by acting as essential precursors (such as FAD and FMN) that support flavoproteins involved in cellular respiration, electron transport, and oxidative stress mitigation [74]. Furthermore, they indirectly support defense systems by modulating carbon metabolism, promoting glutathione biosynthesis, and regulating the activity of key antioxidant enzymes such as SOD, CAT, and GPx [207].

The primary representative compounds include ascorbic acid (vitamin C), which is recognized as one of the most effective water-soluble antioxidants in photosynthetic organisms [211,212]. Additionally, this group comprises several essential B-complex vitamins that exhibit distinct antioxidant roles, namely pyridoxine (vitamin B6), riboflavin (vitamin B2), folate (vitamin B9), and cobalamin (vitamin B12) [30].

Extensive experimental evidence from the literature validates the protective functions and bioactivity of these hydrophilic vitamins in microalgae under adverse environmental conditions. The overall antioxidant potential of microalgal extracts enriched in these water-soluble vitamins has been widely demonstrated through standard in vitro assays, including DPPH, ABTS, and ORAC tests [30,207,209]. Specifically, studies on pyridoxine (vitamin B6) highlight its protective importance in microalgae exposed to external stressors such as high irradiance or nutritional limitation [30,212]. Regarding indirect mechanisms, experimental data confirm that riboflavin (vitamin B2) supports crucial flavoprotein activity [74], while folate (vitamin B9) and cobalamin (vitamin B12) are actively involved in the biochemical pathways of carbon metabolism and glutathione biosynthesis to mitigate oxidative stress [30]. Finally, experimental observations show that these hydrophilic compounds actively enhance cellular defense systems by modulating the specific enzymatic activities of SOD, CAT, and GPx [207].

#### 3.4.4. Current and Prospective Applications of Hydrophilic Vitamins

Microalgae-derived hydrophilic vitamins have attracted interest in applications in the food, pharmaceutical, cosmetic, and aquaculture sectors due to their nutritional value and antioxidant properties [215,216].

Currently, biomass from *Chlorella* and *Arthrospira* species is marketed as a nutritional supplement due to its high content of proteins, vitamins, and antioxidants [216,221]. Microalgae have been investigated as alternative sources of cobalamin (vitamin B12) for the vegetarian and vegan population [206]. However, the presence of non-bioavailable analogs of cobalamin, such as pseudocobalamin, remains an important limitation [218,219].

Vitamin-rich microalgal extracts are increasingly incorporated into cosmetic formulations aimed at reducing oxidative stress and preventing UV-induced photoaging [223]. Similarly, the inclusion of microalgal biomass in aquaculture feeds has been associated with improved growth, immune response, and stress tolerance in farmed marine organisms [224].

Current research focuses on strain selection, optimization of cultivation conditions, metabolic engineering, and photobioreactor design to enhance the commercial viability of microalgae-based vitamin production [222,225]. These approaches may increase vitamin accumulation, reinforcing the role of microalgae as a promising platform for hydrophilic vitamins within biorefinery and blue biotechnology frameworks [217,226].

### 3.5. Phenolic Compounds

Microalgae and cyanobacteria represent a highly promising, sustainable, and economically attractive platform to produce phenolic compounds, particularly flavonoids. These microorganisms do not merely synthesize polyphenols as structural elements, but rather utilize them as a dynamic, non-enzymatic defense mechanism against harsh environmental conditions. The resulting polyphenolic profiles exhibit exceptional antioxidant capacities through coordinated hydrogen atom and single-electron transfer pathways, which may be further enhanced by natural intracellular interactions with carotenoids and endogenous enzymes.

#### 3.5.1. Metabolic Importance of Phenolic Compounds

The metabolic significance of phenolic compounds in microalgae extends beyond antioxidant defense and includes intracellular roles in abiotic stress mitigation, photoprotection, and acclimation mechanisms, as well as allelopathy and cell signaling.

In microalgae, phenolic compounds, including flavonoids, are secondary metabolites that constitute critical components of the non-enzymatic antioxidant defense system [192]. Unlike terrestrial plants, where these compounds heavily contribute to structural integrity (e.g., lignin biosynthesis), microalgal phenolic compounds are primarily synthesized as dynamic physiological responses to environmental fluctuations. The metabolic upregulation of these pathways is tightly linked to the accumulation of ROS within the cell. Under optimal growth conditions, microalgae maintain low basal levels of polyphenols. However, exposure to abiotic stressors, such as UV radiation, high salinity, nutrient limitation (particularly nitrogen and phosphorus), and heavy metals, promotes oxidative stress [73]. To prevent irreversible cellular damage, microalgae activate the phenylpropanoid pathway. These metabolites act as direct ROS scavengers and singlet oxygen quenchers, thereby protecting intracellular structures, including the photosynthetic apparatus and nucleic acids, from lipid peroxidation and enzymatic degradation [227].

#### 3.5.2. Main Species and Phenolic Compounds

Compared with terrestrial plants, microalgae produce specific profiles of simple phenolics, phenolic acids, and flavonoids. The distribution and abundance of these phenolic compounds within microalgae vary extensively across major taxonomic groups, including Chlorophyceae, Bacillariophyceae, and cyanobacteria [73]. Among green microalgae (Chlorophyceae), species of the genera *Chlorella*, *Scenedesmus*, and *Haematococcus* are widely recognized for their high content of polyphenols, particularly when grown under stress conditions [192]. Likewise, the cyanobacterium *Arthrospira platensis* (commercially known as Spirulina) possesses biosynthetic pathways capable of accumulating high concentrations of simple phenolic acids and flavonoids [228]. Several classes of flavonoids, such as flavonols, flavanones, isoflavones, and dihydrochalcones, have been identified in microalgae and cyanobacteria. Furthermore, phenolic acids such as gallic, protocatechuic, caffeic, chlorogenic, vanillic, p-hydroxybenzoic, and salicylic acids have been reported in a wide range of marine microalgal species [229].

Phenolic compounds have two basic chemical structures. The first one is a C6-C1 ring structure, which is a basic skeleton of phenolic acids and hydrolyzed tannins. As the most fundamental non-flavonoid compounds, phenolic acids contain a phenyl ring bound to a carboxyl group and one or more hydroxyl substituents. Moreover, they are further classified as hydroxybenzoic and hydroxycinnamic acids depending on the length of their carbon chain. Conversely, the more complex C6-C3-C6 framework defines flavonoids, condensed tannins, and phenylpropanoid acids. Flavonoids represent the most ubiquitous class of phenolic compounds and feature a characteristic phenyl benzopyran skeleton, where two phenyl rings are linked via a heterocyclic pyran ring. This diverse group is subsequently subdivided into various chemical subclasses, including flavonols, flavones, flavanones, flavanols, isoflavonoids, and anthocyanins, differentiated by their specific pyran ring substitution patterns and bonding configurations [229].

#### 3.5.3. Antioxidant Capacity of Phenolic Compounds

The antioxidant capacity of microalgal phenolic compounds is primarily determined by their molecular structure, which enables them to act as highly efficient free radical scavengers [192]. Unlike isolated synthetic antioxidants, microalgal phenolics act within a complex intracellular antioxidant network [227]. The primary direct mechanism of action involves hydrogen atom transfer (HAT) or single-electron transfer (SET) to scavenge synthetic and organic radicals in the aqueous phases of the cytoplasm and vacuoles. Simultaneously, they can reduce ferric complexes (Fe^3+^) to their ferrous form (Fe^2+^), thereby evaluating the electron-donating capability [45,73,192]. Additionally, phenolic compounds interact with endogenous antioxidant enzymes, such as SOD and CAT, to regenerate oxidized molecules and maintain cellular redox homeostasis under abiotic stress conditions [230].

The primary representative molecules within this category include microalgal phenolics and flavonoids, which are often characterized by high concentrations of specific reference polyphenols such as gallic and caffeic acids [45,73,192]. Within the microalgal biomass, these hydrophilic phenolics operate in tandem with key lipophilic pigments, such as astaxanthin in *Haematococcus lacustris*, β-carotene in *Dunaliella salina*, and fucoxanthin in diatoms, establishing a comprehensive defense matrix across different cellular compartments [73]. Their potential applications and biological sources are summarized in Table 5.

Extensive experimental evidence from studies on extracts from numerous microalgal species has demonstrated that, together with carotenoids, phenolic compounds make a major contribution to the overall antioxidant capacity of microalgal biomass [227]. Evaluating the antioxidant capacity of microalgal extracts requires a combination of standardized in vitro spectrophotometric assays, as a single method cannot capture the diverse chemical nature of the compounds [192]. The most widely utilized assays include total phenolic content, which is quantified using the Folin–Ciocalteu assay, which serves as a baseline indicator of the total reducing capacity of the microalgal extract. Radical scavenging activity is generally assessed by DPPH and ABTS assays, which measure the capacity of the phenolic extracts to scavenge synthetic organic radicals through hydrogen or electron donation, showing a strong positive correlation with high concentrations of gallic and caffeic acids. Ferric reducing antioxidant power (FRAP) measures the specific ability of the polyphenols to reduce a ferric complex (Fe^3+^) to its ferrous form (Fe^2+^), thereby evaluating the electron-donating capability of the extract [45,73,192].

#### 3.5.4. Current and Prospective Applications of Phenolic Compounds

Currently, the commercial viability of phenolic compounds is well-established within high-value niche markets primarily focused on green innovations in the cosmeceutical, nutraceutical, and natural food preservation sectors. The commercial utilization of microalgal phenolic compounds, particularly flavonoids, has expanded rapidly, primarily driven by increasing demand for natural, sustainable, and clean-label ingredients [229].

Microalgal polyphenols are of considerable interest in the cosmetic industry due to their multi-functional skin-protective properties. Extracts from *Chlorella vulgaris* and *Haematococcus lacustris* have been incorporated into commercial anti-aging creams and sunscreens [227]. Moreover, due to their broad therapeutic profile, polyphenols extracted from *Arthrospira platensis* and *Chlorella* are utilized as core ingredients in dietary supplements, functional beverages, and powdered formulations [228]. Clinical studies have correlated regular consumption of these microalgal products with the downregulation of biomarkers for chronic systemic inflammation, improvements in lipid profiles, and protection against cardiovascular diseases through inhibition of LDL oxidation [73].

In the food industry, microalgal phenolic compounds are being investigated as natural alternatives to synthetic lipid antioxidants, such as butylated hydroxytoluene (BHT) and butylated hydroxyanisole (BHA), which face growing scrutiny due to potential health risks [192]. Polyphenolic fractions from chlorophytes have been used to enrich oils and muscle-based food items, effectively retarding lipid oxidation, preventing rancidity, and extending the overall shelf-life of processed food products.

Future research must prioritize the optimization of large-scale cultivation strategies and automated upstream cultivation techniques that trigger maximum phenolic accumulation without compromising overall biomass production. Concurrently, downstream processing must advance toward the integration of multi-product biorefineries utilizing eco-friendly, green extraction solvents to guarantee process sustainability [234]. Finally, overcoming the limitations of low oral bioavailability and chemical instability through advanced nano-encapsulation techniques remains an essential hurdle to unlocking the full pharmaceutical and biomedical potential of microalgal phenolic compounds [235]. Resolving these challenges will further position microalgae as an indispensable source of antioxidant phenolic compounds.

## 4. Sustainable Production of Antioxidant Molecules in Microalgae

The production and downstream purification of these compounds from microalgae is expensive, and current research is focusing on improving their economic feasibility. In this context, several strategies have been explored. One major approach aims to reduce biomass production costs through bioconversion processes based on the “waste to high-value compounds” concept. For this purpose, numerous microalgae have been cultivated in a variety of nutrient-rich effluents, as summarized in Table 6. Notably, most of the studies have been performed with green algae. Chlorophyte microalgae generally exhibit higher tolerance to toxic compounds than other microalgal groups, as exhibited by faster growth rates, making them particularly attractive candidates for the development of circular bioprocesses. Moreover, some species of this group can be readily cultivated under mixotrophic or heterotrophic conditions, which is a key advantage for wastewater-based cultivation systems [236]. Many wastewaters contain high concentrations of organic matter that can be used as carbon, nitrogen, or phosphorus sources for microalgae, enabling simultaneous biomass production and nutrient removal from the contaminated water. In most of the studies summarized in Table 6, removal efficiencies of approximately 90–95% have been achieved after 6–10 days of cultivation. Thus, these waste streams can be effectively converted into microalgal biomass and, consequently, produce antioxidant compounds. However, the use of wastewater in microalgal cultivation also presents some limitations, particularly when the biomass must be used in food, nutraceutical, or pharmaceutical applications. The presence of different pollutants, such as heavy metals, emerging contaminants, or biological pollutants, including viruses and bacteria, may comprise biomass safety and requires adequate monitoring and downstream processes [237,238]. Thus, although wastewater-based cultivation can contribute to reducing freshwater consumption and production costs, its application for high-value antioxidant products requires careful assessment of contaminant risks, product safety, and regulatory requirements.

The production of pigments as high-value compounds is one of the most studied processes in microalgae-based systems. Under a 7-day cultivation period, *Chromochloris zofingiensis* produced 0.71 mg g^−1^ of astaxanthin and 4.04 mg g^−1^ of carotenoids in 10% whey wastewater [239]. On the other hand, *Chlorella sorokiniana* could produce 9.7 mg g^−1^ of lutein after 7 days of cultivation in a 30 L photobioreactor with piggery wastewater [240], demonstrating the capacity to obtain high amounts of this antioxidant compound. Fatty acids are also the main molecules produced by microalgae in wastewater treatment systems. Previous studies performed with *Dunaliella salina* or *Scenedesmus* sp. demonstrated that these strains could produce high amounts of PUFAs, mainly linoleic and linolenic acids, with values of 8.38 and 4.17% of the microalgal dry biomass when cultivated in shrimp aquaculture and municipal wastewater, respectively [241,242].

**Table 6:** Examples of microalgae-based wastewater treatment systems that include antioxidant compound production.

Microalgae	Wastewater	Nutrient Removal (%)			Period (Day)	Produced Compound	Reference
		COD	TN	TP			
*Chlorella sorokiniana*	Piggery wastewater	92	96	97	7	Lutein and α-linolenic acid	[240]
*Chlorella* sp.	Hydroponic effluent	93	95	99	6	PUFAs	[243]
*Chromochloris zofingiensis*	Whey wastewater	86	93	77	7	Astaxanthin and lipids	[239]
*Dunaliella salina*	Shrimp aquaculture wastewater	82	75	-	8	Pigments and fatty acids	[241]
*Neochloris* sp.	Dairy processing wastewater	92	93	63	12	Carotenoids, fatty acids, and polysaccharides	[244]
*Porphyridium purpureum*	Tofu wastewater and glycerol	83	-	-	7	Exopolysaccharides	[245]
*Scenedesmus quadricauda*	Municipal wastewater	83	95	97	12	Fatty acids, polysaccharides, and terpenoids	[246]
*Scenedesmus* sp.	Primary municipal wastewater	76	99	100	10	Fatty acids	[242]

Bioprospecting novel microalgal strains with higher productivity of these compounds than currently identified strains represents an additional strategy for the sustainable production of antioxidant molecules in microalgae. This systematic search for new species plays a key role in the expansion of the microalgal industry [247]. In recent years, microalgal bioprospecting has mainly focused on extreme environments, such as Arctic/Antarctic regions, hot springs, or highly polluted environments [54,248,249]. The interest in these microorganisms, known as extremolytes, has increased lately because of their ability to synthesize unique molecules, which have significant biotechnological potential. The extreme conditions of these environments promote the production of these unique bioactive molecules with strong antioxidant properties [10]. Moreover, these microorganisms can also be cultivated at a large scale with minimal contamination risks due to their tolerance to harsh environmental conditions.

*Galdieria sulphuraria* is one of the most extensively studied extremophilic microalgae. This red microalga can grow under different trophic conditions, at pH values between 0.5 and 3.0 and at temperatures above 56 °C, conditions that facilitate its cultivation at a large scale with a low risk of contamination [250]. Furthermore, its ability to synthesize high amounts of phycocyanin makes it a promising source of antioxidant molecules [251,252]. In a recent study, C-phycocyanin produced by *Galdieria sulphuraria* exhibited similar antioxidant capacity to that produced by *Spirulina platensis* (the current commercial source of phycocyanin) while demonstrating greater thermal stability and photostability. These features make *Galdieria sulphuraria* an alternative source of this antioxidant compound for industrial production [253].

Other extremophilic microalgae also represent remarkable sources of antioxidant compounds. The green microalga *Dunaliella salina*, which can tolerate NaCl concentrations up to 5 M, can accumulate up to 13% of its biomass as β-carotene, compared with the 1–2% of the dry biomass typically produced by other microalgal strains [83]. *Coccomyxa onubensis*, a green microalga isolated in the Tinto River (an ecosystem characterized by high amounts of sulfides and heavy metals and an acidic pH (2–3) [254]), has demonstrated strong antioxidant capacity under heavy metal stress [54], as well as a notable capacity to produce lutein in these conditions [255]. Other examples are *Chlorococcum* sp. and *Scenedesmus* sp., which are two psychrophilic microalgae capable of producing 15.5 and 10.7 mg g^−1^ of lutein and 35 and 27.5 mg GAE g^−1^ of phenolic compounds, respectively, when they are cultivated at low temperatures under high light stress. Moreover, the antioxidant capacity of these microalgae increased at low temperatures of cultivation [42]. *Fistulifera* sp., an alkaliphilic extremophile diatom, is also capable of synthesizing high amounts of fatty acids with antioxidant activity [256], demonstrating that searching for new microorganisms from extreme environments is an excellent approach for finding novel molecules with antioxidant capacities.

Finally, genetic engineering of microalgal strains represents another promising strategy to enhance the production of these compounds. In this context, CRISPR/Cas systems have recently emerged as one of the most powerful genome-editing technologies due to their high precision and efficiency and the absence of regulatory constraints associated with genetically modified organisms (GMOs). This genome-editing tool enables the modification of metabolic pathways through gene knockouts and knockins, thereby promoting the biosynthesis and accumulation of target molecules in microalgae [257].

In recent years, numerous studies have applied CRISPR/Cas technology to microalgae in order to improve the production of antioxidant compounds, particularly carotenoids. Most of these studies were conducted using the model organism *Chlamydomonas reinhardtii*. In this species, Kneip et al. [258] reported a 2.3-fold increase in astaxanthin production following the knockout of the *lycopene ε-cyclase* gene. Similarly, Jang et al. [108] achieved a 190-fold increase in zeaxanthin content compared with the parental strain through the knockout of the *zeaxanthin epoxidase* and *lycopene ε-cyclase* genes. In another study, Molina-Márquez et al. [259] obtained 7.8 mg g^−1^ of phytoene in this microalga by disrupting the *phytoene desaturase* gene. Another microalgal species widely used for modifications of the carotenoid biosynthetic pathway is *Phaeodactylum tricornutum*. In this diatom, the knockout of the cryptochrome resulted in a 1.3-fold increase in fucoxanthin content [260]. Additionally, Volpe et al. [261] reported a 7-fold increase in diatoxanthin levels after knocking out the *zeaxanthin epoxidase 3* and *chloroplast signal recognition particle 54* genes in this species.

Other antioxidant compounds whose production has been enhanced in microalgae using CRISPR/Cas technology include fatty acids. Ryu et al. [262] reported a 4-fold increase in linoleic acid and a 1.5-fold increase in EPA after knocking in the *Δ12-fatty acid desaturase* gene at the T1 locus of *Nannochloropsis salina* CCMP1776. Similarly, Guo et al., [263] achieved a 1.7-fold increase in lignoceric acid levels by knocking down the *enoyl-CoA hydratase* gene in *Phaeodactylum tricornutum*.

In addition to CRISPR/Cas systems, other genetic engineering strategies have been optimized in microalgae to enhance the production of different bioactive molecules. Li et al. [264] reported a 15-fold increase in tocopherol content in *Chlamydomonas reinhardtii* through chloroplast transformation with a codon-optimized *homogentisate phytyltransferase vitamin E2* gene from *Synechocystis*. In another study, Zeng et al. [265] obtained a 1.6-fold increase in DHA content by using atmospheric and room-temperature plasma mutagenesis in the microalga *Schizochytrium*. Moreover, increases of 1.5-fold in EPA levels were reported by Miao et al. [266] in *Nannochloropsis oceanica* through the overexpression of the *NoDGAT2K* gene driven by the *NO22G01450* promoter/terminator. Genetic engineering also enables the simultaneous enhancement of multiple target compounds. In this regard, Liu et al. [267] increased canthaxanthin and EPA production by 20- and 1.3-fold, respectively, after introducing the *β-carotenoid ketolase* gene from *Chlamydomonas reinhardtii* into the chloroplast of *Nannochloropsis oceanica*. These results highlight the considerable potential of genetic engineering approaches for boosting the production of antioxidant compounds in microalgae.

## 5. Conclusions and Future Perspectives

Altogether, microalgae represent a highly promising and sustainable source of antioxidant compounds with biotechnological potential. The different metabolites discussed in this review, some of which are unique to specific microalgal taxa, have demonstrated high antioxidant activity, as well as a range of additional bioactive properties. While the biosynthesis of certain compounds appears to be constitutive within microalgal metabolism, including lutein, EPA, biotin, and gallic acid, the accumulation of other metabolites, such as astaxanthin, β-carotene, and ascorbic acid, can be markedly enhanced under specific environmental stress conditions. Consequently, the selection of an appropriate microalgal strain, together with the optimization of cultivation strategies, is crucial for increasing the production of targeted high-value antioxidant compounds and improving the overall efficiency of microalgal bioprocesses.

Thus, microalgae constitute a sustainable and versatile platform to produce antioxidant and other bioactive compounds, often providing molecules with high-purity and naturally occurring bioactive isomers that may be difficult to obtain through chemical synthesis. Despite their potential, the large-scale commercialization of microalgae-derived antioxidants remains constrained by scientific and technological bottlenecks. These include the trade-off between biomass productivity and antioxidant accumulation, the variability of metabolite production among strains and cultivation conditions, the incomplete characterization of some biosynthetic pathways, and the high costs associated with harvesting and downstream processing. Among the compounds discussed in this work, carotenoids such as astaxanthin, lutein, β-carotene, and fucoxanthin represent particularly promising targets because of their high antioxidant activity, market value and commercial relevance, although their production and preservation still present technological challenges. Consequently, future research should prioritize the selection and development of high-performing strains; optimized cultivation; two-stage production systems, as developed for *Dunaliella salina* and *Hameatococcus lacustris*; and, where appropriate, metabolic engineering and omics-guided approaches to enhance productivity while maintaining biomass yields. Furthermore, advances in downstream processing, including greener extraction technologies, improved stabilization and formulation strategies, and the valorization of multiple biomass fractions within integrated biorefinery platforms, will be essential to reduce production costs and enhance product quality and process sustainability. Finally, standardization of antioxidant activity, bioaccessibility, product safety, and regulatory requirements to facilitate the translation of laboratory-scale findings into commercially viable food, nutraceutical, cosmetic, and pharmaceutical applications should be addressed. Overall, these advances will be essential to accelerate the integration of microalgae into industrial biotechnology, reinforcing their role as a key resource for the sustainable production of antioxidant molecules within emerging blue biotechnology biorefinery frameworks.

## Figures and Tables

**Table 2:** The most abundant PUFA with more than 3 unsaturations is described in the main classes of microalgae. Data represent general behavior; composition varies depending on species and culture conditions. ALA: α-linolenic acid; SDA, stearidonic acid; OPA, octadecapentaenoic acid; ARA: arachidonic acid; EPA: eicosapentaenoic acid; DHA: docosahexaenoic acid.

Microalgae Group	Characteristic PUFA (≥3 Double Bonds)	Comments	References
Chlorophyceae	ALA, 16:4 ω3	Mainly PUFA C16-C18; EPA and DHA in low amounts	[22,124,125]
Trebouxiophyceae	ALA	Profile dominated by PUFA C18	[22,124,125]
Prasinophyceae	ALA, SDA, OPA, EPA, DHA (some species) and	Especially rich in ω3; OPA is a characteristic biomarker	[22,126]
Bacillariophyceae (diatoms)	EPA and ARA	Highly enriched in EPA	[22,127,128]
Eustigmatophyceae	EPA	Significant EPA producers	[22,125,127]
Cryptophyceae	ALA, 18:4 ω3, EPA, and DHA	High nutritional value, simultaneous presence of EPA and DHA	[22,124,128]
Haptophyta (Prymnesiophyceae)	SDA, EPA, and DHA	Significant producers of DHA	[117,127,129]
Dinophyceae	OPA, DHA, and EPA	Significant producers of DHA	[117,127,129]
Thraustochytriaceae (*Schizochytrium* and *Aurantiochytrium*)	DHA	Significant producers of DHA	[117,130,131]

**Table 5:** Summary of major phenolic compounds from microalgae: functions, applications, and biological sources.

Phenolic Compound	Main Microalgal Sources	Potential Applications	References
Gallic acid	*Spirulina platensis* and *Chlorella vulgaris*	Food preservation and antimicrobial agents	[192,231]
Caffeic acid	*Haematococcus lacustris* and *Isochrysis galbana*	Anti-inflammatory drugs and UV-protective cosmetics	[45,73]
Ferulic acid	*Tetraselmis suecica* and *Chlorella pyrenoidosa*	Anti-aging formulations and polymer stabilization	[74]
Catechin	*Dunaliella salina* and *Scenedesmus obliquus*	Cardioprotective nutraceuticals and functional foods	[45,192]
Quercetin	*Phaeodactylum tricornutum* and *Chlorella vulgaris*	Dietary supplements and immune boosters	[192,232]
Resveratrol	*Spirulina platensis*	Biomedical therapies and life-span extension supplements	[233]

## Data Availability

No new data were created or analyzed in this study. Data sharing is not applicable to this article.

## Abbreviations

The following abbreviations are used in this manuscript:

ABTS	2,2-azino-*bis*-3-ethylbenzothiazoline-6-sulphonic acid
ALA	α-linolenic acid
APX	Ascorbate peroxidase
ARA	Arachidonic acid
BHA	Butylated hydroxyanisole
BHT	Butylated hydroxytoluene
CAT	Catalase
DHA	Docosahexaenoic acid
DPA	Docosapentaenoic acid
DPPH	1,1-diphenyl-2-picrylhydrazil
EPA	Eicosapentaenoic acid
FAD	Flavin adenine dinucleotide
FAs	Fatty acids
FAS	Fatty acids synthase complex
FMN	Flavin mononucleotide
FRAP	Ferric reducing antioxidant power
GLA	γ-linolenic acid
GMOs	Genetically modified organisms
GPx	Glutathione peroxidase
GR	Glutathione reductase
GSH	(Reduced) glutathione
GSSG	Oxidized glutathione
GST	Glutathione-S transferase
NCDs	Non-communicable diseases
OPA	Octadecapentaenoic acid
ORAC	Oxygen radical absorbance capacity
Prxs	Peroxiredoxins
PUFAs	Polyunsaturated fatty acids
ROS	Reactive oxygen species
SDA	Stearidonic acid
SOD	Superoxide dismutase
TAC	Total antioxidant capacity
TBARSs	Thiobarbituric acid reactive substances
